# Cell migration and antigen capture are antagonistic processes coupled by myosin II in dendritic cells

**DOI:** 10.1038/ncomms8526

**Published:** 2015-06-25

**Authors:** Mélanie Chabaud, Mélina L. Heuzé, Marine Bretou, Pablo Vargas, Paolo Maiuri, Paola Solanes, Mathieu Maurin, Emmanuel Terriac, Maël Le Berre, Danielle Lankar, Tristan Piolot, Robert S. Adelstein, Yingfan Zhang, Michael Sixt, Jordan Jacobelli, Olivier Bénichou, Raphaël Voituriez, Matthieu Piel, Ana-Maria Lennon-Duménil

**Affiliations:** 1 Inserm U932, Institut Curie, 26 rue d'Ulm, 75248 Paris cedex 05, France; 2 CNRS UMR144, Institut Curie, 26 rue d'Ulm, 75248 Paris cedex 05, France; 3 CNRS UMR3215/Inserm U934, Institut Curie, 26 rue d'Ulm, 75248 Paris cedex 05, France; 4 Laboratory of Molecular Cardiology, National Heart, Lung, and Blood Institute, National Institutes of Health, Bethesda, Maryland 20892, USA; 5 Institute of Science and Technology Austria, Am Campus 1, 3400 Klosterneuburg, Austria; 6 National Jewish Health & University of Colorado, 1250 14th Street, Denver, USA; 7 CNRS UMR 7600, Université Pierre et Marie Curie, 4 Place Jussieu, 7600 Paris, France; 8 CNRS FRE 3231, Université Pierre et Marie Curie, 4 Place Jussieu, 75005 Paris, France

## Abstract

The immune response relies on the migration of leukocytes and on their ability to stop in precise anatomical locations to fulfil their task. How leukocyte migration and function are coordinated is unknown. Here we show that in immature dendritic cells, which patrol their environment by engulfing extracellular material, cell migration and antigen capture are antagonistic. This antagonism results from transient enrichment of myosin IIA at the cell front, which disrupts the back-to-front gradient of the motor protein, slowing down locomotion but promoting antigen capture. We further highlight that myosin IIA enrichment at the cell front requires the MHC class II-associated invariant chain (Ii). Thus, by controlling myosin IIA localization, Ii imposes on dendritic cells an intermittent antigen capture behaviour that might facilitate environment patrolling. We propose that the requirement for myosin II in both cell migration and specific cell functions may provide a general mechanism for their coordination in time and space.

## Abstract (web summary)

Dendritic cells alternate between fast and slow migratory behaviours, however in the absence of a component of the antigen processing machinery, migration is uniform and fast. Chabaud *et al*. now show that slow migration results from the relocalisation of myosin II to the cell front where it promotes antigen capture.

## Graphical abstract

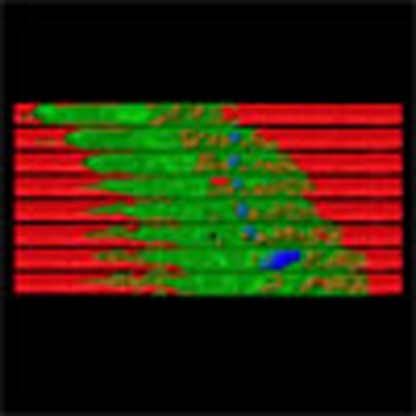

Dendritic cells (DCs) are in charge of capturing antigens in peripheral tissues, transporting them to lymph nodes and presenting them on major histocompatibility complex (MHC) molecules to T lymphocytes. This process referred to as antigen presentation leads to T-cell activation and is essential for the onset of the adaptive immune response. In tissues, immature DCs capture antigens mainly by phagocytosis and macropinocytosis^1^. This actin-dependent mode of internalization allows the nonspecific uptake of large amounts of extracellular fluid and, in DCs, relies on the small GTPases Cdc42 and Rac1 (refs 2, 3). Taken-up antigens are delivered to endolysosomes, where they are degraded into peptides to be loaded on MHC class II molecules^4^.

How immature DCs uptake antigens *in vivo* to exert their patrolling function has recently started to be documented. Two-photon imaging experiments suggest that in certain tissues, such as the mouse ear and gut, DCs randomly migrate to scan the environment^5^,^6^. In contrast, in the mouse footpad and lung, DCs were shown to rather remain sessile and uptake luminal antigens through membrane projections that cross the epithelia^7^,^8^,^9^,^10^. Whether these different DC behaviours rely on cell-intrinsic mechanisms that allow the coordination between their antigen capture function and their migratory capacity remains unknown.

The mechanisms that regulate DC migration are not fully understood. An essential role was attributed to the actin-based motor protein myosin II. Its activity is required both *ex vivo* and *in vivo* for migrating DCs to reach their maximal speed in three-dimensional (3D) environments^11^,^13^. Integrin-dependent adhesion was found to be dispensable to this process^11^. Using microfabricated channels that mimic the confined space of peripheral tissues, we have shown that the MHC class II-associated invariant chain (Ii or CD74) regulates the motility of immature DCs by imposing transient phases of slow locomotion^12^. In addition, myosin IIA and Ii were found to physically interact in both DCs and B cells^12^,^14^. However, neither the mechanism by which Ii reduces DC locomotion nor the impact of such regulation on the antigen capture function of DCs has been highlighted so far.

Here we show that antigen capture and DC migration both require myosin IIA. Efficient antigen uptake by macropinocytosis is associated with periodic enrichments of Myosin IIA at the DC front. These enrichments disrupt the back-to-front myosin IIA gradient responsible for fast locomotion and therefore slow down cell speed. These results indicate that there is a cell-intrinsic antagonism between fast cell migration and antigen uptake. We further show that this antagonism relies on the regulation of myosin IIA localization by Ii, which is required for the recruitment of the motor protein at the front of DCs. We propose that this migration mode imposes on immature DCs a migratory behaviour that might facilitate their ability to detect scattered antigens, as suggested by a model based on intermittent search optimization.

## Results

### Myosin IIA is enriched at the DC front during slow migration

We aimed at understanding how myosin II controls the migration of immature DCs in confined environments. We have previously shown that in microchannels their speed is compromised by the addition of the myosin II inhibitor Blebbistatin^12^. A similar speed reduction was observed when analysing bone-marrow-derived DCs differentiated from conditional knockout mice for myosin IIA, the only myosin II isoform expressed in mouse DCs (Immunological Genome project http://www.immgen.org) (Supplementary Figs 1a–e). No further decrease was observed on Blebbistatin treatment, excluding a compensatory effect by myosin IIB or IIC (Supplementary Fig. 1e). Migration of *myosin IIA*^*−/−*^ immature DCs on epidermal ear sheets was equally decreased (Supplementary Fig. 1f,g). We conclude that, as shown for other types of cells, the migration of immature DCs in confined environments such as microchannels or the interstitial space of tissues depends on myosin II-driven contractile forces^15^.

**Figure 1:**
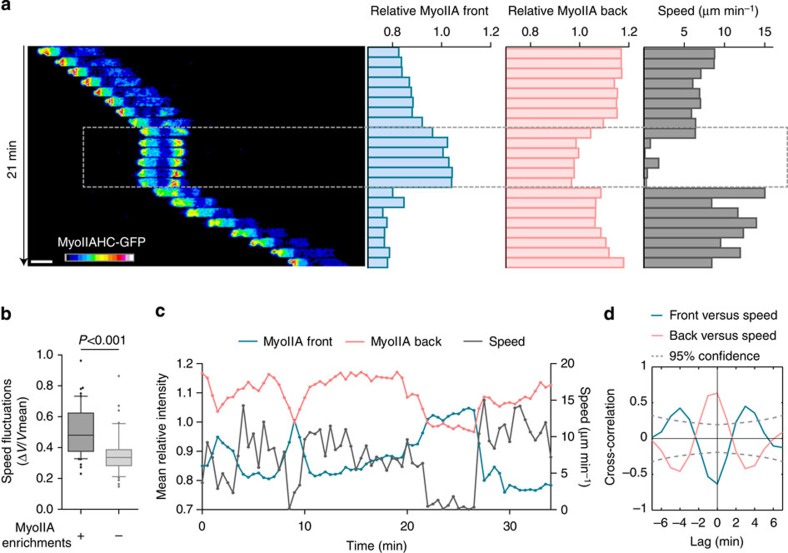
Myosin IIA is enriched at the front of DCs during slow motility phases. (**a**) Sequential epifluorescence images (× 20, one image every minute) of a myosin IIA-GFP knock-in DC exhibiting one event of myosin IIA-GFP enrichment at the cell front: the average intensity of myosin IIA-GFP at the front (1) and the back (2) relative to the average intensity of the entire cell were measured together with the cell instantaneous speed (obtained by tracking the nucleus centre of mass; 3). Scale bar, 10 μm. (**b**) Quantification of speed fluctuations (calculated as s.d./mean instantaneous speed^12^ in DCs displaying or not myosin IIA-GFP enrichments at the front within 15 min (*n*=50 cells and 44 cells, respectively, three independent experiments). Data are represented as box and whiskers (10–90 percentile) plus outliers. A Mann–Whitney test was applied for statistical analysis. (**c**) Layered curves of the three parameters described in (**a**) in a cell undergoing three phases of myosin IIA-GFP enrichment at the cell front. (**d**) Mean cross-correlation values obtained from the three parameters measured in (**a**) in 20 cells that underwent at least one event of myosin IIA-GFP enrichment at the front during motion (two independent experiments).

As previously reported^12^,^13^, immature DCs display significant speed fluctuations during motion. To investigate the role of myosin IIA in this peculiar migration mode, we characterized its dynamics in DCs differentiated from myosin IIA heavy chain-green fluorescent protein (GFP) knock-in mice^16^. We observed that phases of slow motion were associated with transient events of myosin IIA enrichment at the DC front (Fig. 1a, amplitude, duration and frequency quantified in Fig. 3d–f). Accordingly, migrating DCs that displayed myosin IIA enrichments at their front during the observation period also underwent more speed fluctuations (Fig. 1b). In contrast, myosin IIA was mostly located at the DC rear during phases of fast motility (Fig. 1a,c and Supplementary Movie 1). To quantify this phenomenon, we measured the levels of myosin IIA at the cell rear and front and evaluated whether these parameters were temporally cross-correlated with the speed of DCs. This analysis showed that slow motility phases systematically correlated with myosin IIA enrichment at the DC front (Fig. 1d, blue curve) and myosin IIA reduction at the DC rear (Fig. 1d, pink curve). Hence, myosin IIA localization at the rear of DCs correlates with fast migration, whereas myosin IIA enrichment at the front of DCs is associated with slow locomotion.

### Myosin IIA enrichment at the DC front slows down migration

**Figure 2:**
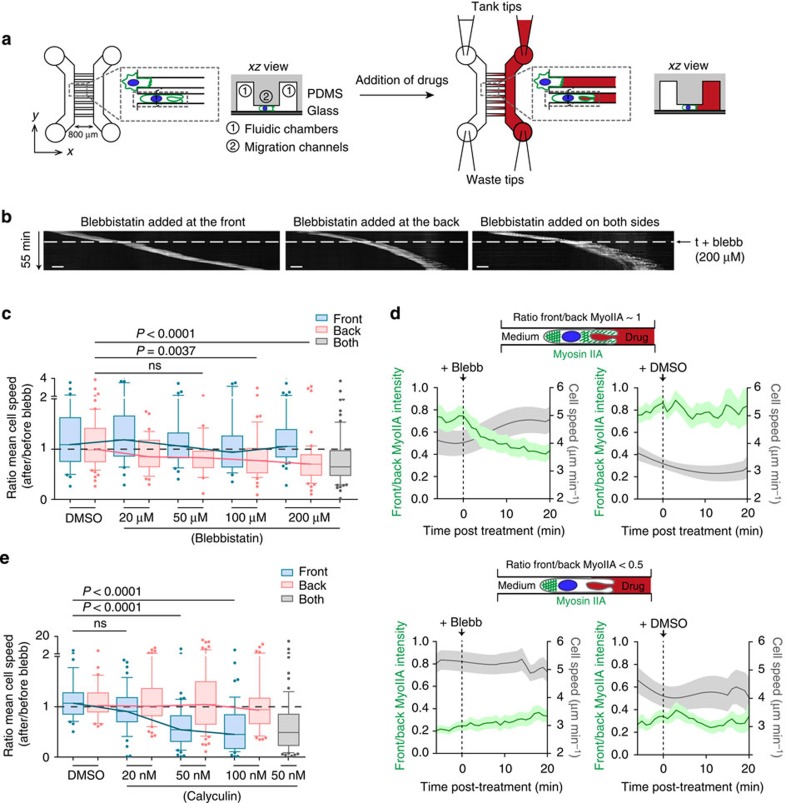
Myosin IIA enrichment at the front of DCs reduces their speed of locomotion. (**a**) Schematic of the microfluidic device used to deliver drugs at the front or rear of DCs, composed of migration microchannels (5 × 5 μm) in between two fluidic chambers. Parallel flows were obtained by filling the ‘tanks tips' (with medium-containing drugs or not) but leaving the ‘waste tips' emptied, leading to the formation of a perpendicular gradient along migration channels. Clogging of a migration channel by one cell resulted in an on/off drug delivery. (**b**) Representative kymographs of cells receiving 200 μM Blebbistatin at *t*=15 min either at their front (left), rear (middle) or on both sides (right; × 10, one image every 30 s during 55 min). Scale bar, 20 μm. (**c**) Ratio of the mean instantaneous speed measured before and after delivery of Blebbistatin. Data from four independent experiments are represented as box and whiskers (10–90 percentile) and outliers (30–70 cells per condition). A Kruskal–Wallis test was applied for statistical analysis. (**d**) Smoothed instantaneous speed versus ratio of (total myosin IIA-GFP intensity at the cell front)/(total mosin IIA-GFP intensity at the cell rear) before and after delivery of 50 μM para-nitroblebbistatin (non-phototoxic blebbistatin, left panels) or DMSO (right panels) at the front of migrating myosin IIA-GFP knock-in immature DCs. Cells were classified in two groups: cells that have high amount of myosin IIA-GFP at their front (upper panels) or cells that have almost no myosin IIA-GFP at their front (lower panels) before treatment (*n*=18–20 cells per condition from four independent experiments). (**e**) Ratio of the mean instantaneous speed measured before and after delivery of Calyculin A. Data from four independent experiments are represented as box and whiskers (10–90 percentile) and outliers (30–70 cells per condition). A Kruskal–Wallis test was applied for statistical analysis.

These data suggest that myosin IIA localization at the DC rear promotes fast motility, whereas its enrichment at the DC front slows down migration. To test this hypothesis, we built a microfluidic device in order to selectively modulate myosin II activity at the rear or front of migrating DCs and evaluated the impact of such modulation on their speed. For this, we simultaneously injected culture medium in one chamber and drug-containing medium in the opposite chamber, resulting in the formation of a gradient in the microchannels that connect them (Fig. 2a and Supplementary Fig. 2a, upper panel). Clogging of microchannels by one cell impaired gradient formation but resulted in the selective delivery of the drug to one cell pole (Supplementary Fig. 2a, lower panel)^17^.

Addition of increasing concentrations of Blebbistatin at the DC rear reduced their mean instantaneous velocity in a dose-dependent manner and to a similar extent than inhibition of myosin II on both cell poles (Fig. 2b,c). Addition of Blebbistatin at the front of DCs did not significantly alter their speed at the level of the entire cell population. However, we consistently observed that some DCs that had received Blebbistatin at their front exhibited decreased speed fluctuations that correlated with increased cell velocity (Supplementary Fig. 2b). We reasoned that this differential effect of Blebbistatin might depend on whether myosin II was enriched or not at the cell front at the time of drug addition. To test this hypothesis, we used a non-phototoxic form of Blebbistatin (para-nitroblebbistatin), which impairs cell migration (Supplementary Fig. 2c) and is compatible with GFP imaging. We observed that addition of this compound during phases of myosin IIA-GFP enrichment at the cell front markedly increased the migration speed of DCs (Fig. 2d, upper left panel, grey curve). This result indicates that myosin IIA activity at the cell front indeed impairs cell migration. Noticeably, this acceleration was accompanied by a reduction in the pool of myosin IIA-GFP located at the cell front (Fig. 2d, upper left panel, green curve). No such effects were observed in DCs that did not show myosin IIA-GFP enrichment at their front at the time of drug addition (Fig. 2d, lower left panel) or in DCs treated with dimethylsulphoxide (DMSO; Fig. 2d, upper and lower right panels). Hence, myosin IIA inhibition at the cell rear compromises DC migration, whereas myosin IIA inhibition at the DC front promotes fast motility.

To strengthen these results, we performed similar experiments using Calyculin A, a phosphatase inhibitor that promotes myosin II activity by increasing the phosphorylation levels of its regulatory light chain (myosin light chain (MLC); Supplementary Fig. 2d)^18^. Calyculin A had no effect when applied at the DC rear (Fig. 2e), suggesting that local contractility cannot be further increased. In contrast, addition of Calyculin A at the front of DCs strongly decreased their speed, showing that activation of myosin II at the DC front compromises their locomotion. Altogether, our results indicate that (1) fast migration relies on the pool of myosin IIA located at the rear of DCs and (2) slow migration results from the enrichment of the motor protein at their front. We therefore conclude that speed fluctuations observed in migrating immature DCs result from the repeated disruption of the front-to-back gradient of myosin IIA because of recruitment of the motor protein to the cell front.

### Enrichment of myosin IIA at the DC front requires li

**Figure 3:**
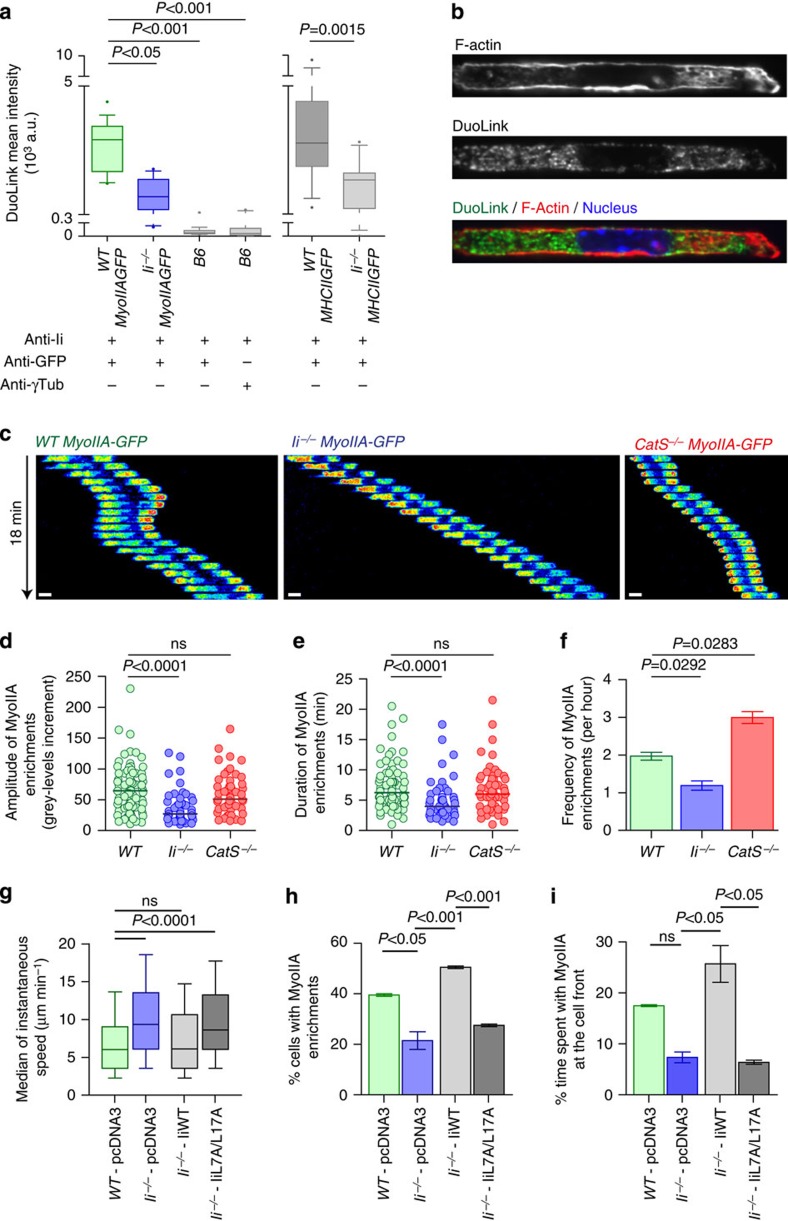
Myosin IIA enrichment at the DC front is promoted by Ii. (**a**) Quantification of the Duo-Link signal mean fluorescence intensity on the entire cell using different DC types and antibody combinations, as indicated. Data are represented as box and whiskers (10–90 percentile) plus outliers. *n*=12–17 cells per condition from one representative experiment out of three. A Kruskal–Wallis and a Mann–Whitney test was applied for statistical analysis of left and right panels, respectively. (**b**) Spinning disk images (× 100, middle plane) of a fixed immature WT myosin IIA-GFP knock-in DC after PCR-based Duo-Link amplification using anti-Ii and anti-GFP antibodies. Scale bar, 5 μm. (**c**) Sequential epifluorescence pseudocolour images (× 20, one image every min), of representative myosin IIA-GFP knock-in WT, Ii^*−/−*^ and CatS^*−/−*^ DCs. Scale bars, 10 μm. (**d**) Amplitude (grey level increment) and (**e**) duration of myosin IIA enrichments in myosin IIA-GFP knock-in WT, Ii^*−/−*^ and CatS^*−/−*^ DCs (*n*=80, 48 and 55 cells, respectively, four independent experiments). Bars represent the medians. Littermate controls were used. A Kruskal–Wallis test was applied for statistical analysis. (**f**) Number of myosin IIA enrichments per hour, in myosin IIA-GFP knock-in WT, Ii^*−/−*^ and CatS^*−/−*^ DCs (*n*=224, 174 and 122 cells, respectively, from eight independent experiments). A paired *t*-test was applied for statistical analysis. (**g**) The median of instantaneous speed of WT or Ii^*−/−*^ bone marrow DCs transfected on day 6 of differentiation with an empty vector (WT-pcDNA3), full-length human Ii (Ii^*−/−*^-IiWT) or human Ii whose leucines 7 and 17 were replaced by alanines (Ii^*−/−*^-IiL7AL17A). Data from three independent experiments are represented as box and whiskers (10–90 percentile). *n*=430 cells per condition. A Kruskal–Wallis test was applied for statistical analysis. (**h**) Percentage of DCs exhibiting at least one event of myosin IIA-GFP enrichment at their front during 1 h of recording and (**i**) percentage of time spent with myosin IIA-GFP at the front of myosin IIA-GFP knock-in DCs transfected as described in **g**. Data from two independent experiments are shown (*n*=53 WT-pcDNA3, 36 Ii^*−/−*^-pcDNA3, 55 Ii^*−/−*^-IiWT and 67 Ii^*−/−*^-IiL7AL17A cells). One-way analysis of variance (ANOVA) Bonferroni tests were applied for statistical analysis.

We aimed at understanding how myosin IIA is recruited at the DC front. We have previously shown that (1) myosin IIA interacts with Ii in both B cells and DCs^12^,^14^ and (2) Ii-deficient immature DCs exhibit less speed fluctuations as compared with wild-type cells^12^. We therefore hypothesized that myosin IIA recruitment at the DC front, which triggers phases of slow motion, might be regulated by Ii. Consistent with this hypothesis, structure illumination microscopy (SIM) images revealed a partial co-localization of myosin IIA heavy chain and Ii at both the cell rear and front of DCs (Supplementary Fig. 3a and Supplementary Movie 2). This co-localization was quantified using the PCR-based ‘Proximity Ligation Assay', or ‘Duo-Link'^19^. A strong positive signal was obtained when labelling GFP and Ii in myosin *IIA-GFP* DCs (*WT MyoIIAGFP* on Fig. 3a,b), indicating that the two proteins are located at a distance inferior to 50 nm. This signal was similar to the one measured when staining for GFP and Ii molecules in MHC class II-GFP knock-in DCs, MHC class II and Ii being known to directly associate (*WT MHCIIGFP* on Fig. 3a). Conversely, Duo-Link signals were strongly reduced in DCs that do not express Ii or myosin IIA-GFP (*Ii*^*−/−*^ *MyoIIAGFP* and B6 on Fig. 3a). The specificity of the Ii–myosin IIA interaction was further supported by the negative signal obtained with the cytosolic protein gamma-tubulin (Fig. 3a). Quantification of the Duo-link-positive signal showed that Ii and myosin IIA interacted at both the rear and front of DCs (Supplementary Fig. 3b), confirming the observations made using SIM.

Having shown that the two proteins physically associate, we investigated whether Ii regulates myosin IIA localization during DC migration. For this, we monitored myosin IIA dynamics in DCs expressing different levels of Ii: DCs knock out for Cathepsin S (CatS), which are known to accumulate Ii because of impaired proteolysis^20^ and Ii-deficient DCs (Fig. 3c and Supplementary Movie 1). We found that the amplitude, the duration and the frequency of myosin IIA enrichments were all significantly decreased in Ii-deficient DCs (Fig. 3d–f). In addition, the percentage of Ii-silenced DCs showing events of myosin IIA enrichments at their front within 15 min was lower as compared with control DCs (Supplementary Fig. 3c–e), excluding a bystander effect of Ii gene deletion. These data are consistent with our previous study showing that *Ii*^*−/−*^ cells display less speed fluctuations during motion^12^. In contrast to *Ii*^*−/−*^ DCs, myosin IIA enrichment events were more frequent in *CatS*^*−/−*^ DCs, but their amplitude and duration were comparable to the ones of wild-type (*WT*) DCs (Fig. 3d–f). These results strongly suggest that Ii is required for transient myosin IIA enrichments at the front of migrating DCs. In agreement, we found that ectopic expression of human Ii (Ii-p33) in mouse *Ii*^*−/−*^ DCs reduced their speed to *WT* levels (Fig. 3g) and restored myosin IIA recruitment at their front (Fig. 3h,i). Strikingly, this was not observed when using the IiL7A/L17A Ii mutant (Fig. 3g–i), which was previously shown to remain at the cell surface instead of reaching endolysosomal compartments (Supplementary Fig. 3f)^21^. Consistent with this result, although both WT Ii and IiL7A/L17A mutant were expressed at similar levels when transfected in DCs, only WT Ii-p33 and its cleavage intermediates Ii-p25 and p16 were pulled down with endogenous myosin IIA-GFP (Supplementary Fig. 3g). We conclude that Ii triggers the periodic recruitment of myosin IIA at the DC front and thereby leads to important speed fluctuations.

### Myosin IIA at the DC front regulates macropinosome dynamics

**Figure 4:**
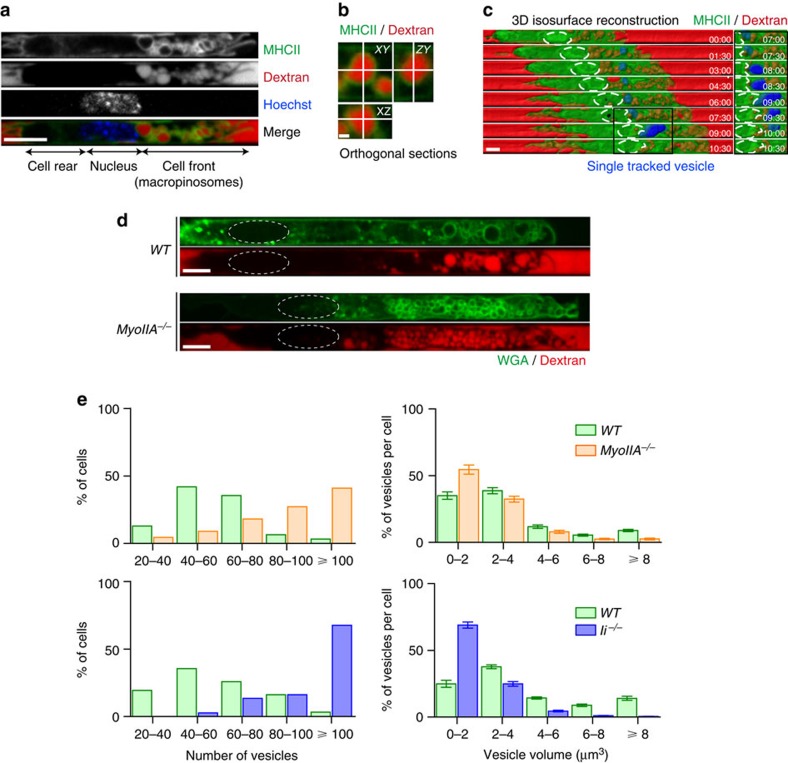
Enrichment of myosin IIA at the front of DCs regulates macropinosome formation. (**a**) Spinning disk image (× 60, middle plane) of a live MHCII-GFP knock-in immature DC migrating in a microchannel filled with 10 kDa AF647-Dextran. The nucleus was stained with Hoechst. (**b**) Orthogonal sections of a dextran-containing vesicle. Scale bar, 1 μm. (**c**) 3D isosurface reconstruction from a sequential 3D stack of 10 images with 0.5 μm *z*-step (× 60, one image every 30 s) of an MHCII-GFP immature DC migrating in a microchannel filled with 10 kDa AF647-Dextran. The nucleus position is indicated with dotted lines. A single vesicle is highlighted in blue for trafficking visualization, progressively switching from shade blue (when inside the cell, that is, below the green signal) to bright blue (once exposed outside). Zoomed images show an exocytotic event taking place in front of the nucleus. (**d**) Spinning disc images (× 60, middle plane) of live WT and myosin IIA^*−/−*^ immature DCs (stained with 10 μg ml^−1^ AF488-WGA for membrane visualization) migrating in microchannels filled with 10 kDa AF647-Dextran. (**e**) Distribution of vesicle numbers (left panels) and volumes (right panels) in myosin IIA^*−/−*^ and WT DCs (31 and 23 cells), Ii^*−/−*^ and WT DCs (31 and 37 cells). Littermate controls were used. Cells were pooled from eight independent experiments and displayed altogether to overcome the limited amount of cells per experiment. Scale bars, 5 μm except when indicated.

As the main function of immature DCs is to sample their environment by engulfing large amounts of extracellular material, we next investigated whether the pool of myosin IIA observed at the DC front plays a role in antigen uptake. For this, we took advantage of our microfluidic device that allows introducing and rapidly exchanging fluorescent antigens in microchannels (Supplementary Fig. 4a) in order to follow and quantify the sequential steps leading to antigen internalization: vesicle formation (Supplementary Fig. 4b) and antigen arrival to endolysosomes (Supplementary Fig. 4c). We observed giant vesicles filled with extracellular antigens that formed at the front of DCs, whether fluorescent dextran or ovalbumin (OVA) were used (Fig. 4a,b and Supplementary Fig. 4b). Washout experiments combined to lysosome staining showed that the fluorescent antigens that were retained inside the cell had been delivered to endolysosomes, where antigen processing takes place (Supplementary Fig. 4c). The actin-depolymerizing agent Latrunculin A and the macropinocytosis inhibitor 5-(*N*-ethyl-*N*-isopropyl)amiloride (EIPA)^22^ or impaired antigen uptake at the cell front, indicating that antigen-containing vesicles correspond to macropinosomes (Supplementary Fig. 4d). Such vesicles were not observed in migrating neutrophils, demonstrating that they were not a bystander product of confinement inside microchannels but rather resulted from the strong macropinocytic capacity of DCs (Supplementary Fig. 4e). Interestingly, tracking of single macropinosomes at high temporal resolution in wild-type DCs showed that they emptied part of their content to the extracellular milieu when reaching the area in front of the nucleus (see blue vesicle on Fig. 4c and Supplementary Movie 3). This is consistent with previous findings showing that macropinocytosis is associated with exocytic events that allow membrane recycling and prevent cell volume increment^23^.

**Figure 5:**
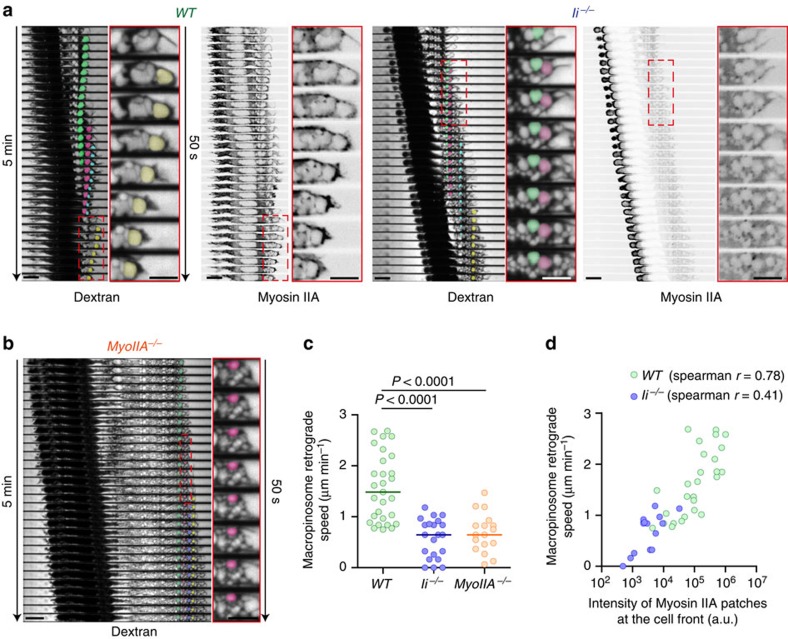
Ii-dependent recruitment of myosin IIA to the front of DCs regulates macropinosome dynamics. (**a**) Sequential spinning disc images (× 60, middle plan, one image every 10 s) of myosin IIA-GFP knock-in WT and Ii^*−/−*^ immature DCs migrating in microchannels filled with 10 kDa AF647-Dextran. (**b**) Sequential spinning disc images (× 60, middle plan, one image every 10 s) of a myosin IIA^*−/−*^ immature DC migrating in a microchannel filled with 10 kDa AF647-Dextran. (**a**,**b**) Single macropinosomes were colour-coded to follow their intracellular transport. (**c**) Macropinosome velocity in WT, Ii^*−/−*^ and myosin IIA^*−/−*^ DCs. Bars show the medians (*n*=29 WT, 21 Ii^*−/−*^ and 17 myosin IIA^*−/−*^ from two independent experiments). A Kruskal–Wallis test was applied for statistical analysis. (**d**) Correlative graph of macropinosome retrograde speed versus myosin IIA patches intensity at the cell front (*n*=28 WT and 22 Ii^*−/−*^ from two independent experiments). WT DCs correspond to Ii^+/+^ DCs (littermate controls). Scale bars, 5 μm.

To define whether the pool of myosin IIA recruited to the DC front has an impact on macropinosome formation during DC migration, we analysed the dynamics of these vesicles in immature myosin IIA-deficient DCs. *Myosin IIA*^*−/−*^ DCs displayed macropinosomes that were considerably increased in number but reduced in volume (Fig. 4d,e). Similar results were obtained when analysing Ii-deficient DCs, in which myosin IIA is not efficiently recruited at the cell front (Fig. 4e). Noticeably, characterization of macropinosome dynamics showed that they underwent a rearward displacement in *WT* cells, whereas they were rather static in both myosin *IIA*^*−/−*^ and *Ii*^*−/−*^ DCs (Fig. 5a,b and Supplementary Movie 4). Accordingly, measurement of macropinosome velocity revealed that it was significantly reduced in both cell types as compared with *WT* cells (Fig. 5c), indicating that myosin IIA enrichment at the DC front is required for intracellular macropinosome trafficking towards the cell rear. Consistent with these observations, a strong correlation between the amount of myosin IIA-GFP present at the cell front and the retrograde velocity of macropinosomes was observed (Fig. 5d). Interestingly, we found that myosin IIA enrichment at the DC front was concomitant to events of cell front shortening (Fig. 5a), suggesting that macropinosome retrograde transport might result from local myosin II-mediated contraction. We conclude that myosin IIA regulates macropinosome dynamics at the DC front by promoting (1) the formation of large macropinosomes and (2) macropinosome retrograde intracellular transport.

### Myosin IIA facilitates antigen delivery to endolysosomes

**Figure 6:**
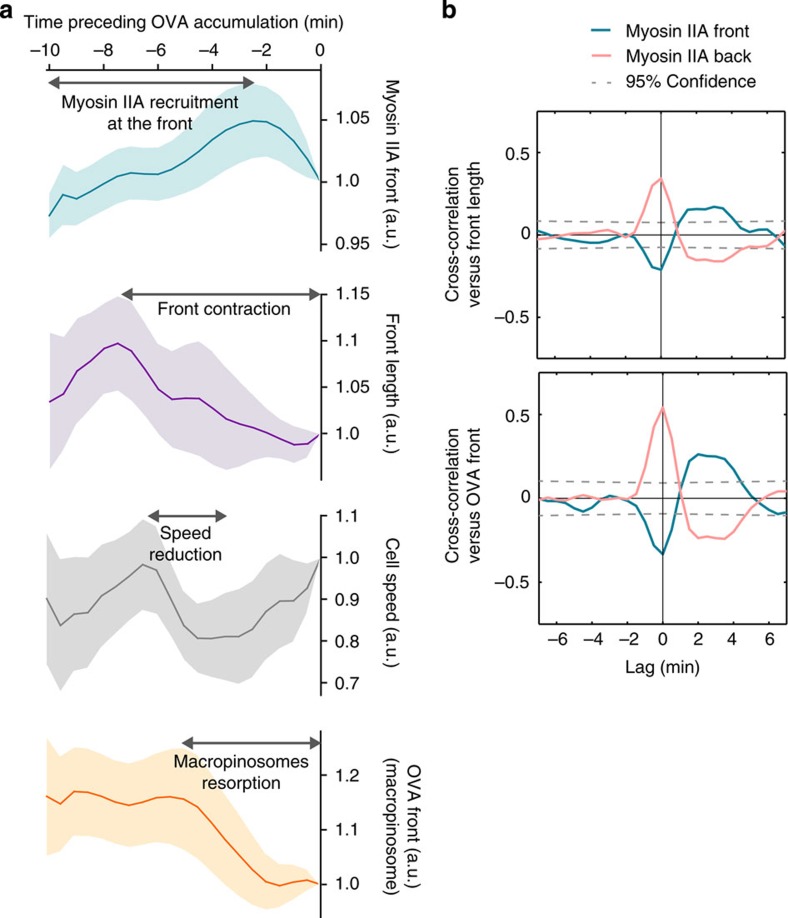
Myosin IIA enables antigen accumulation by promoting cell front contraction. (**a**) Relative curves representing the mean intensity of myosin IIA-GFP signal at the DC front, the DC front length, the speed of DCs and the total intensity of fluorescent OVA signal at the DC front. Data were obtained during the 10 min preceding the arrival of OVA to endolysosomes, from 12 cells showing at least one event of myosin IIA-GFP at their front (three independent experiments). Data are represented as mean±s.e.m. (**b**) Mean cross-correlation values observed between the myosin IIA-GFP mean intensity at the cell front and back, the front length and the total intensity of the fluorescent OVA signal measured at the cell front. Data were obtained from 23 cells showing at least one event of myosin IIA-GFP at their front (four independent experiments).

We next investigated whether the involvement of myosin IIA in macropinosome dynamics regulates subsequent events of antigen transport to endolysosomes. As shown above, these compartments, where antigens are ultimately delivered and processing takes place, are located behind the nucleus at the rear of DCs (Supplementary Fig. 4c). To address this question, we simultaneously monitored myosin IIA enrichment at the cell front, antigen arrival into endolysosomes and cell speed (Supplementary Fig. 5a,b). We observed that after antigen loading into microchannels the first peak of antigen arrival to endolysosomes was preceded by a cascade of events starting with progressive myosin IIA enrichment at the DC front (−10 min), cell front contraction (−8 min, as measured by front length), cell velocity decrease (−7 min) and macropinosome resorption (−5 min; Fig. 6a and Supplementary Fig. 5c). In addition, cross-correlation analyses showed that cell front shortening systematically correlated with maximal myosin IIA enrichment at the DC front (Fig. 6b, top panel) as well as with macropinosome disappearance (Fig. 6b, bottom panel). Together, these results suggest that myosin IIA enrichment at the front of DCs leads to local contraction, an event that has two consequences: (1) it reduces cell speed and (2) it promotes macropinosome resorption and antigen delivery to endolysosomes.

**Figure 7:**
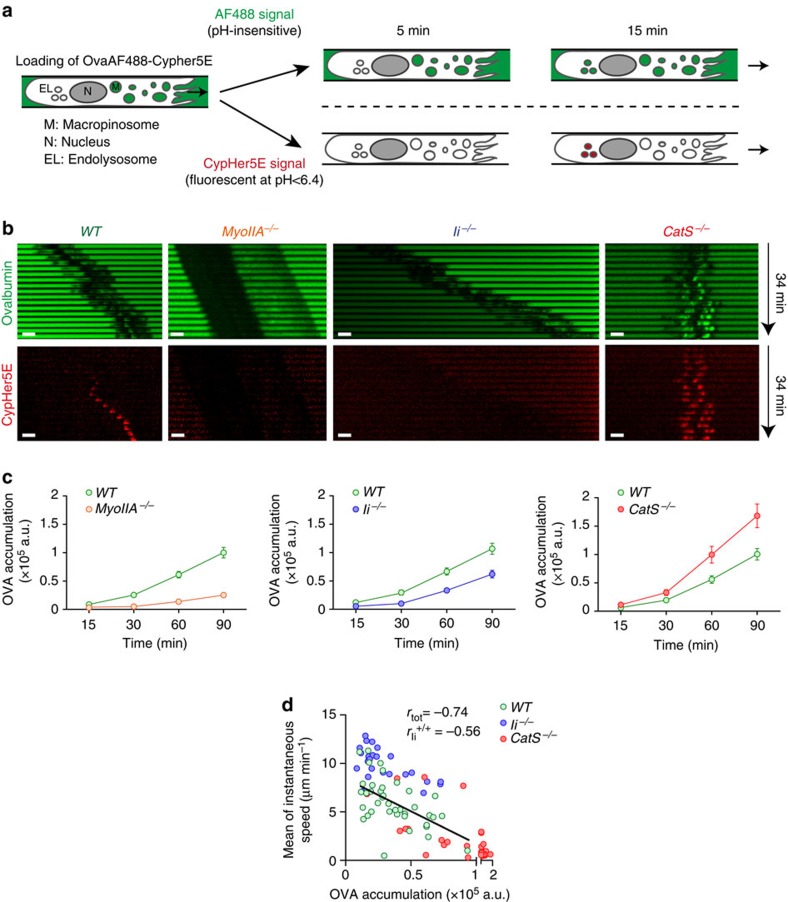
Regulation of macropinosome dynamics by myosin IIA facilitates antigen transport to endolysosomes. (**a**) Experimental design to monitor antigen transport to endolysosomes using OVA coupled to both AF488 (green) and CypHer5E (red). Arrows indicate the direction of migration. (**b**) Sequential wide field microscopy images (× 20, one image every 2 min) of WT, myosin IIA^*−/−*^, Ii^*−/−*^ and CatS^*−/−*^ immature DCs migrating along microchannels filled with AF488-OVA-CypHer5E. Scale bars, 10 μm. (**c**) OVA accumulation into endolysosomes in migrating myosin IIA^*−/−*^ versus WT DCs (left), Ii^*−/−*^ versus WT DCs (middle) and CatS^*−/−*^ versus WT DCs (right) immature DCs, quantified as AF488-OVA sum intensity accumulated into CypHer5E-positive compartments normalized by the amount of OVA taken up at the cell front, represented as mean±s.e.m. Cells were pooled from two to three independent experiments (>50 cells per condition, WT DCs correspond to littermate controls). (**d**) Graph showing a correlation between the final levels (*t*=90 min) of AF488-OVA in endolysosomes and the mean of cell instantaneous velocities.

To confirm the role of myosin IIA in antigen transport, we monitored the kinetics of antigen arrival into endolysosomal compartments in DCs that exhibit different levels of myosin IIA at their front. For this, we labelled the OVA antigen with two dyes: (1) pH-insensitive AlexaFluor-488 and (2) Cypher5E, which fluoresces at a pH below 6.4 (refs 24, 25; Fig. 7a). Antigen arrival to endolysosomes, monitored by measuring the AlexaFluor-488 fluorescence in Cypher5E-positive compartments, was visible 10–15 min on its addition to the extracellular milieu and linearly increased during the 90 min of recording (Supplementary Fig. 6a). This antigen accumulation was inhibited by EIPA, indicating that it strictly relied on macropinocytosis (Supplementary Fig. 6b). Strikingly, the ability of *myosin IIA*^*−/−*^ DCs to accumulate taken-up antigens into endolysosomes was strongly impaired as compared with *WT* DCs (Fig. 7b,c, left panel and Supplementary Movie 5). *Ii*^*−/−*^ DCs, which display low levels of myosin IIA at their front, also exhibited a defective antigen accumulation capacity (Fig. 7b,c, middle panel and Supplementary Movie 5). This is unlikely to result from a global endocytic defect in *Ii*^*−/−*^ DCs since levels and distribution of endocytic markers, endosomal pH and ability to transport the transferrin receptor CD71 to endolysosomes were not altered in these cells (Supplementary Fig. 6c–h). Hence, myosin IIA enrichment at the DC front promotes antigen arrival into endolysosomal compartments. In contrast to Ii knockout DCs, the ability of CatS-deficient DCs to deliver antigens to endolysosomes was significantly increased (Fig. 7b,c, right panel and Supplementary Movie 5). We further observed that the final levels of antigen accumulation and the mean instantaneous velocity of DCs were inversely correlated (Fig. 7d): while most *Ii*^*−/−*^ DCs clustered in the upper left part of the graph, indicating that their increased velocity was associated with impaired antigen accumulation, *CatS*^*−/−*^ DCs clustered in the lower right part of the graph, showing that their reduced migration was associated with enhanced antigen capture. Altogether, our data show that, while myosin IIA localization at the cell rear promotes fast migration of DCs, its Ii-dependent transient enrichments at the cell front reduce cell speed but allow efficient antigen capture, resulting in the coordination of these two processes in time and space.

### Immature DCs behave as intermittent searchers

We have shown that immature DCs alternate phases of fast and slow locomotion, the latter being associated with efficient antigen capture. Interestingly, this biphasic migration mode is reminiscent of ‘intermittent random walks', which were shown to minimize the search time of randomly located targets^26^. The intermittent random walk model, which so far proved to be applicable in various contexts, from target search by proteins on DNA to animal behaviour^26^,^27^,^28^,^29^, relies on the assumption that efficient probing of the environment by an agent looking for a target is incompatible with fast motion: search trajectories are in that case characterized by an alternation of slow motion with high detection abilities, and fast motion with reduced detection. This feature qualitatively fits our observations and makes the intermittent random walk model a natural framework to analyse the search efficiency of immature DCs.

**Figure 8:**
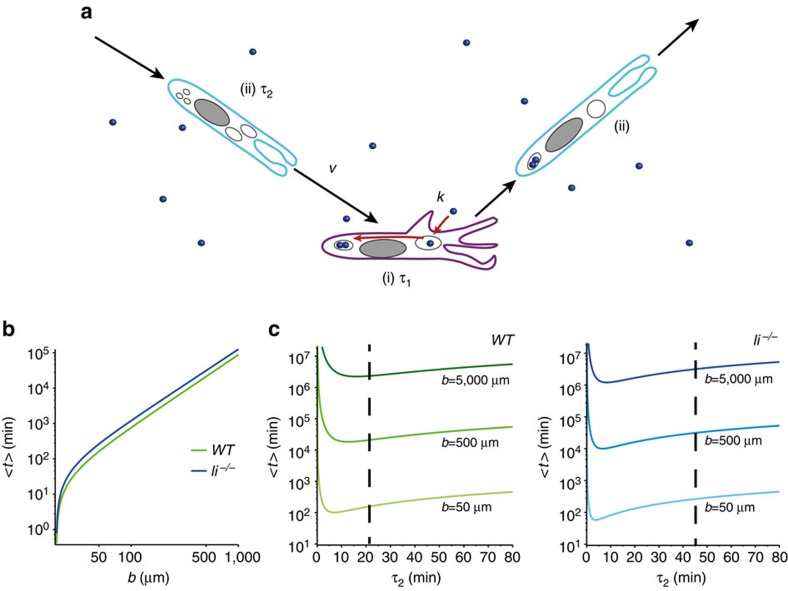
Ii optimizes the search of antigens by DCs. (**a**) DC migration as an intermittent random walk: the intermittent random walk model assumes that DCs alternate between two migration phases (i) and (ii). In the slow phase (i; purple cell), of mean duration *τ*_1_, cell motion is neglected and antigen (blue particles) capture occurs at rate *k* (in all plots we consider the regime *k>>1* of efficient antigen uptake). In the fast phase (ii; blue cell) of mean duration *τ*_2_, antigen capture is neglected and cell velocity is *v.* (**b**) Antigen mean search time <*t*> (log scale) expressed as a function of the mean distance *b* between antigen locations. Cell capture radius *a*=20 μm, all other parameters were determined from data shown in Fig. 3. WT: *v*=3.9 μm min^−1^, *τ*_1_=7.8 min, *τ*_2_=22.2 min. li^*−/−*^: *v*=8 μm min^−1^, *τ*_1_=5 min, *τ*_2_=45 min. (**c**) Mean search time for an antigen expressed as a function of *τ*_2_ for different antigen concentrations. Dashed lines highlight the values measured for *τ*_2_. Parameters as in (**b**).

For the sake of simplicity, we consider a DC migrating in a two-dimensional environment, where antigens are randomly distributed and interspersed with a mean distance *b* that parameterizes their concentration. The DC is assumed to alternate slow migration phases (i) during which efficient antigen uptake occurs, and fast migration phases (ii) during which antigen uptake is neglected. The mean durations of phases (i) and (ii) are denoted as *τ*_1_ and *τ*_2_; the velocity in phase (ii) has a random direction and modulus *v*, whereas motion in phase (i) is neglected in a first approximation (Fig. 8a). Following ref. 26, the efficiency of the search process can be quantified by the mean search time *<t>* for a target, which can be obtained analytically (see Supplementary Methods). Using the values of the parameters extracted from cell track analysis and myosin IIA-GFP dynamics (Fig. 3d–f), we obtained a quantitative determination of the mean search time (Fig. 8b,c and Supplementary Methods). *τ*_1_ and *τ*_2_ could either be estimated from the analysis of myosin IIA-GFP enrichment at the cell front (Fig. 3d–f) or from using a threshold value of 3 μm min^−1^ to define the slow migration phases, below which DCs efficiently uptake antigens (Fig. 7d), giving similar results. This analysis yielded a mean search time *<t>∼*15 h for a single cell, and for a mean distance *b*=100 μm (WT DCs). It showed that *WT* cells perform 30% faster than Ii knockout DCs for a broad range of antigen concentrations (Fig. 8b). As expected from the intermittent random walk model, we found that *<t>* could be minimized by adjusting the duration *τ*_2_ of fast migration phases (Fig. 8c and Supplementary Fig. 7). Notably, the experimental value of *τ*_2_ was found to lie near the theoretical optimum, which only weakly depends on *b*, for WT DCs. In contrast, it was significantly larger for *li*^*−/−*^ DCs, indicating that these cells ‘waste time' in long fast phases of locomotion. This model suggests that the observed intermittent behaviour of immature DCs is kinetically close to optimal for antigen capture in 1D microchannels.

## Discussion

In this work, we show that myosin IIA is transiently and repeatedly recruited to the front of migrating immature DCs, an event that promotes efficient antigen capture but slows down locomotion. How does myosin IIA enrichment at the cell front reduce DC velocity? Migration under confinement was proposed to be favoured by a pressure gradient that is strongly enhanced by myosin IIA back–front polarization. This model predicts that generation of a contractile force at the cell front should reduce this pressure gradient and thereby lead to a decrease in cell velocity^30^. Our microfluidic data showing that myosin IIA activation at the DC front decreases DC velocity strongly support this hypothesis. This implies that the loss of speed observed on recruitment of myosin IIA at the cell front does not result from a competition for myosin IIA between front and back but rather from the loss of the myosin IIA activity gradient required for fast locomotion.

We show that efficient myosin IIA enrichment at the front of DCs requires Ii, consistent with our previous findings demonstrating that Ii-deficient DCs migrate faster and exhibit less velocity fluctuations than wild-type DCs^12^. We have previously shown that myosin IIA and Ii form a protein complex in DCs and B cells^12^,^14^. In addition, super-resolution microscopy as well as Duo-Link experiments indicate that Ii and myosin IIA are in close proximity at the cell front, even though their association occurs all along the cell body. Ii might facilitate the recruitment of myosin IIA at the DC front through a direct protein–protein interaction or more indirectly, for example, by locally regulating actin dynamics or myosin II assembly^31^. Our findings that the IiL7A/L17A Ii mutant, which was shown to remain at the cell surface instead of reaching endosomes^21^, did neither associate with myosin IIA-GFP nor recruit the motor protein at the DC front suggest that the interaction between both proteins might take place in endolysosomal compartments. Alternatively, Ii Leucines 7 and 17 might be involved in its association to the motor protein. Interestingly, it was recently shown that the production of PiP_3_ at the front of polarized amoebas promotes macropinocytosis but compromises chemotaxis^32^. Whether Ii facilitates myosin IIA recruitment at the DC front through a direct protein interaction or by modifying local signalling remains to be explored.

The involvement of myosin IIA in macropinocytosis is consistent with observations made by others showing that myosin II is recruited to membrane ruffles in other cell types and that inhibition of MLC phosphorylation compromises the ability of macrophages to engulf particles^33^,^34^. We found that enrichment of myosin IIA at the DC front allows the retrograde transport of macropinosomes, indicating that contractile forces provided by the motor protein might be required for macropinosomes to be transported inwards and for their content to reach endolysosomes. Accordingly, cross-correlation analyses indicate that progressive myosin IIA recruitment at the DC front ultimately leads to cell front contraction and macropinosome resorption. These events are followed by the arrival of antigen into endolysosomes, suggesting that myosin IIA-dependent cell front contraction allows the delivery of their antigenic content to endolysosomes. The exact step at which myosin IIA acts to favour antigen delivery from macropinosomes to endolysosomes remains to be identified. Interestingly, we recently showed that myosin II polarity and activity are controlled by the release of intracellular calcium from the ER through IP3 receptors^13^. Whether ER calcium also controls macropinosome formation and antigen transport to endolysosomes shall now be investigated.

Our work shows that the main function of DCs—antigen internalization for processing and presentation to T cells—is coupled to their migratory capacity and antagonizes it. We propose that coupling between macropinocytosis and DC migration enables the coordination of these two processes in time and space. These results are consistent with findings showing that DC activation transiently increases antigen uptake but reduces their motility^35^. They are further supported by *in vivo* two-photon imaging data showing that in the skin, lung and gut extracellular antigens are internalized by non-motile DCs^6^,^7^,^10^. We further show that the antagonism between locomotion and antigen uptake in immature DCs fulfils the conditions of applicability of the intermittent random walk model. Although our analysis, which relies on parameters extracted from experiments in a one-dimensional environment, would need to be confirmed by further *in vivo* studies, it nonetheless indicates that the migratory behaviour of immature DCs might facilitate their antigen search capacity.

In conclusion, this work addresses the unexplored fundamental question of how cells adapt their migratory behaviour to efficiently perform their effector function(s). We show that, in immature DCS, the coupling of cell function to cell migration results from their common requirement for myosin II. As myosin II controls the motility of many cell types^36^,^37^,^38^,^39^, we foresee that involvement of this motor protein in other cellular processes (for example, polarity or gradient sensing) might lead to a coupling of these processes to cell locomotion.

## Methods

### Mice

Ii and CatS knockout mice as well as I-Aβ^b^-GFP (referred to as MHC II-GFP) and myosin IIA heavy Chain-GFP knock-in mice (referred to as myosin IIA-GFP) were previously described^12^,^16^. Myosin IIA knockout mice were generated by crossing *MyoIIAflox/flox* mice^40^ with *CD11c-Cre* mice^41^ and with *Gt(ROSA)26Sor–flox-stop-flox–YFP* mice (*Rosa-YFP*)^42^ to obtain *MyoIIAflox/flox-CD11c-Cre+-Rosa-YFP* mice that were used as bone marrow donors. Littermates or sex- and age-matched *MyoIIAwt/wt-CD11c-Cre+-Rosa-YFP* mice were used as control bone marrow donor mice. The breeder mice were previously backcrossed to *C57BL/6* for at least seven generations. The experiments were performed on 6-week-old male or female mice. For animal care, we strictly followed the European and French National Regulation for the Protection of Vertebrate Animals used for Experimental and other Scientific Purposes (Directive 2010/63; French Decree 2013-118). The present experiments, which used mouse strains displaying non-harmful phenotypes, did not require a project authorization and benefited from guidance of the Animal Welfare Body, Research Centre, Institut Curie.

### Cells

Mouse bone marrow cells were cultured during 10–12 days in medium supplemented with fetal calf serum and granulocyte–macrophage colony-stimulating factor-containing supernatant obtained from transfected J558 cells, as previously described^12^. To generate neutrophils, we differentiated PLB985 (kindly provided by P. Lutz) with All-trans Retinoic Acid as previously described^43^. HEK293Ts were maintained in culture as recommended by the manufacturer (American Type Culture Collection).

### Antibodies and reagents

The following reagents were used for imaging experiments: CFSE and CMTMR (Molecular Probes), Dextran AlexaFluor-647, Ovalbumin AlexaFluor-488, WGA AlexaFluor-488 or -647, Hoechst (Life Technologies), CypHer5E (GE Healthcare) and pHRhodo Red SE (Life Technologies). For drug treatments, we used EIPA (Life Technologies), Latrunculin A (Calbiochem), Blebbistatin (Tocris Bioscience), Para-nitroblebbistatin (Optopharma Ltd), Calyculin A (Sigma), Concanamycin A (Tocris) and DMSO (VWR). We coated the microchannels with Fibronectin (Sigma). PLB985s were differentiated with All-trans Retinoic Acid (Sigma). For immunofluorescence, we used anti-myosin IIA Heavy Chain (Covance, 1/500), AlexaFluor-coupled Phalloïdin (Invitrogen, 1/200), Atto-425-coupled Phalloidin (Sigma, 1/200), anti-Ii IN1 (BD Bioscience, 1/50), anti-GFP (Living Colors, Clontech, 1/200), anti-γTubulin (kind gift from M.Bornens, 1/1,000), anti-Lamp1 (BD Bioscience, 1/500), anti-LYVE-1 (R&D System, 1/1,000), endosomal markers anti-Clathrin HC (1/100), anti-EEA1 (1/100), anti-Rab11(1/100) and anti-Rab7 (1/50; Cell Signaling kit), anti-CD71 (Thermo Scientific, 1/50) and anti-human Ii (PIN1 and BU45 from cell supernatant, 1/2). Slides were mounted with Fluoromount-G (Southern Biotech). For immunoblot, we used anti-phospho-MLC Thr18/Ser19 (Cell signaling, 1/1,000), anti-myosin II (Abcam and ECM Bioscience, both at 1/1,000), anti-myosin IIA Heavy Chain (Covance, 1/1,000), anti-Ii IN1 (BD Bioscience, 1/50), anti-actin (Millipore, 1/1,000) and anti-α-tubulin (Serotec, 1/1,000) and a homemade purified rabbit mAb anti-human Ii raised against C-term (C1C2, 1/10). For flow cytometry, we used a homemade 24G2 anti-Fc Receptor MoAb, rabbit serum from Agro Bio as a control and the following antibodies: anti-MYH9 (Covance, 1/250), anti-CD11c (HL3 clone, 1/100) and anti-I-Aβ^b^ (AF6-120.1 clone, 1/100). For lentivirus production, HEK cells were transfected using GeneJuice (Novagen).

### DC imaging on epidermal ear sheets

Epidermal ear sheets were removed from the ear ventral part, as previously described^44^. They were stained by adding anti-LYVE-1 in the culture medium for 1 h at room temperature. In the meantime, *WT* and *myosin IIA*^*−/−*^ DCs were stained with CFSE 5 μM and CMTMR 5 μM at 10 millions per ml in PBS for 20 min at 37 °C, incubated for 5 min at 37 °C in serum and washed twice in the culture medium. Overall, 20,000 cells of each cell type were mixed and loaded on the stained epidermal sheet. After 4 h at 37 °C, epidermal sheets were washed twice with the culture medium to remove residual cells in suspension that did not penetrate the tissue. Epidermal sheets were imaged using a spinning disk microscope consisting of Yokagawa CSU-X1 spinning head mounted on an Eclipse Ti inverted microscope (Nikon) equipped with a Coolsnap HQ2 camera (Photometrics) with a × 20 numeric aperture (NA) 0.75 taking one image every 2 min, on a thickness of 100 μm.

### Preparation of microchannels and speed quantification

Microchannels were prepared as previously described^12^,^45^. Briefly, microfluidic devices were fabricated in polydimethylsiloxane using rapid prototyping and soft lithography. PDMS pieces and glass-bottom fluorodish (World Precision Instruments) were activated in a plasma cleaner (PDC-32G Harrick) for 30 s and were stuck altogether. They were then incubated with 20 μg ml^−1^ fibronectin for 1 h and washed with PBS. For velocity quantification, cells were loaded into microchannels and imaged during 20 h on an epifluorescence video-microscope Nikon TiE microscope equipped with a cooled CCD (charge-coupled device) camera (HQ2, Photometrics) with a × 10 objective, taking one transmission phase image every 2 min. Kymograph extraction and instantaneous velocity analysis were performed using a homemade programme as described previously^12^.

### Microfluidics for local drug delivery

Microchannels^45^ with 5 × 5 μm section and a length of 800 μm were used to deliver drugs at the rear or front of cells. In all, 10^5^ cells per well were loaded and allowed to enter microchannels overnight. The following day, P1000 tips were placed in each well, tips were filled with medium±drugs (tank tips) on one side or left emptied on the other side (waste tips) to create a flow. During the first 15 min, medium alone was loaded into the tank tips filling the microfluidic chambers. It was then removed and replaced by medium alone on one chamber and drug-containing medium on the opposite chamber. This resulted in the formation of a gradient in the microchannels that connect the two fluidic chambers. Cells were recorded during 40 min post loading. Clogging of microchannels by one cell impaired gradient formation but resulted in the selective delivery of the drug to one cell side. Migrating cells were imaged using a wide field video-microscope Nikon TiE microscope equipped with a cooled CCD camera (HQ2, Photometrics) with a × 10 Ph 0.3 NA. Cell instantaneous velocities were calculated for each cell before and after drug delivery. To avoid artefacts induced by flow, the first 5 min post loading were not considered for speed measurements. The effect of drugs was assessed by calculating the ratio between the mean of instantaneous speeds measured before and after drug treatment. For experiments with para-nitroblebbistatin, we used myosin IIA-GFP knock-in immature DCs. Total myosin IIA intensity corrected for background was measured by drawing rectangles at the cell front and at the cell rear for each time point. The ratio of front/back total myosin IIA intensity was calculated. In parallel, instantaneous speeds were obtained by tracking the rear of cells and were smoothed using a floating window of three time points.

### Macropinocytosis dynamics in migrating DCs

Microchannels were modified to facilitate the diffusion of dyes and drugs^45^. Microchannel section was 5 × 5 μm and the channels had a length of 350 μm. Immature *Lifeact-GFP* DCs were incubated for 30 min in 200 ng l^−1^ Hoechst-containing medium, washed and 10^5^cells per well were loaded and incubated overnight to allow their entry into microchannels. The following day, treatments for macropinocytosis inhibition were performed by injecting increasing concentrations of EIPA (20, 50 and 100 μM) 2 h before image acquisition or increasing concentrations of Latrunculin A (100, 250 and 500 nM) just before acquisition. Next, 1 mg ml^−1^ of 10 kDa AlexaFluor647-Dextran was loaded in the drug-containing medium and cells were imaged during 2 h using a wide field video-microscope Nikon TiE microscope equipped with a cooled CCD camera (HQ2, Photometrics) with a × 20 Ph 0.75 NA. The volume of dextran internalized by each cell was quantified as dextran present inside the cell front mask given by Lifeact-GFP signal in front of Hoechst signal.

For high-resolution imaging, I-Aβ^b^-GFP knock-in cells were imaged using a spinning disk microscope consisting in a Yokagawa CSU-X1 spinning head mounted on an Eclipse Ti inverted microscope (Nikon) equipped with a Coolsnap HQ2 camera (Photometrics) with a × 60 objective. 3D stacks and isosurface reconstruction were obtained using the Imaris 7.2.3 software, allowing the quantification of object volume and number.

### OVA delivery to endolysosomes in migrating DCs

AlexaFluor-488 OVA was coupled to CypHer5E, which fluoresces below pH=6.4. Coupling was performed following the manufacturer's manual (GE Healthcare). pH sensitivity of the dye was checked by measuring the optical density at pH=7.4 in PBS compared with pH=5.5 in CH_3_COOH using a NanoDrop (Labtech). Fluorescence of CypHer5E was only detected at acidic pH. The microfluidic device used for this experiment was equivalent to the one used in macropinocytosis assays. In all, 10^5^ cells per well were loaded and left overnight to allow their spontaneous entry into microchannels. Just before image acquisition, OVA coupled to AlexaFluor-488 and CypHer5E was added, and cells were immediately imaged using an epifluorescence video-microscope Nikon TiE microscope equipped with a cooled CCD camera (HQ2, Photometrics) with a × 10 or × 20 objective during 90 min. Movies were quantified using either Imaris 7.2.3 or the Integrated Morphometry Analysis tool of Metamorph (Molecular Devices). The CypHer5E signal was used as a mask and applied on the AF488-OVA signal at different time points. The amount of OVA accumulated in endolysosomes was normalized by the amount of OVA taken up at the cell front. To address the impact of macropinocytosis inhibition on the ability of cells to accumulate OVA, DCs migrating along microchannels were pre-treated with 50 μM EIPA for 2 h. To correlate OVA delivery to endolysosomes along time to cell instantaneous speed, manual tracking of single cells was performed using the tracking point plugin of Metamorph.

### Cross-correlation analysis

Myosin IIA HC-GFP knock-in DCs were stained, when indicated, with Hoechst (200 ng ml^−1^) and AlexaFluor555-WGA (5 μg ml^−1^) for 15 min in culture medium at 2 × 10^6^ ml^−1^. After two washes with culture medium, they were loaded in microchannels. After 12 h, cells were imaged at low resolution with an epifluorescence video-microscope Nikon TiE microscope equipped with a cooled CCD camera (HQ2, Photometrics) with a × 20 objective. When indicated, AlexaFluor647-OVA at 0.5 mg ml^−1^ was added to the channels just before acquisition. Data collection for cross-correlation analysis was carried out using the Metamorph software (Molecular Devices). Front and back regions were determined using myosin IIA-GFP signal relative to the position of the nucleus. Front and back myosin IIA relative intensities correspond to the average intensity of each region divided by the average intensity of the entire cell after background subtraction. Accumulation of AlexaFluor647-OVA in endolysosomes was measured in the AlexaFluor555-WGA mask. Cell speed was calculated from the position of the nucleus. The normalized cross-correlation functions between discrete signals *f*(*t*) and *g*(*t*) (either speed or fluorescence signal of cells) represent the mean of the normalized cross-correlation function of individual cells 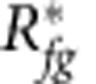, which is calculated as follow:


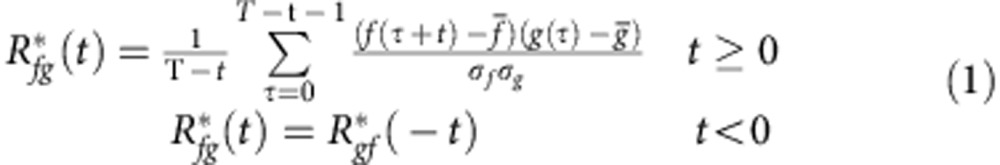


where *T* is the length of the signals and *σ* is the s.d. The 95% confidence threshold *C*(*t*) of correlation coefficients is approximated from the number of products involved in the correlation coefficients and corrected from the autocorrelation of signals using the following expression:


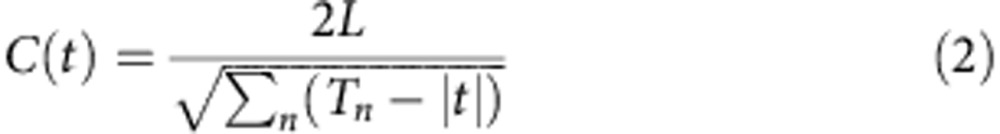


For all cells *n*, where *L* is the maximum of autocorrelation lengths between signals *f* and *g.*

### Semiautomatic analysis of myosin IIA-GFP enrichments

Myosin IIA enrichments were analysed using a custom routine written in Matlab (Mathworks) as following. First, to avoid any noisy detection, a low-pass filter with a cutoff frequency of three sampling steps was applied to the raw data (myosin IIA mean intensity at the front). The initiation of myosin IIA-GFP enrichment events was detected when the derivative signal became positive. Enrichment amplitude was calculated as the difference in grey levels between the highest value and the lowest value of the myosin IIA-GFP mean front intensity. To exclude enrichments due to steady-state fluctuations of myosin IIA at the front, an amplitude threshold of 10 grey levels was applied. All enrichments automatically detected with this routine were ultimately confirmed by eye.

### Macropinosome retrograde transport

Myosin IIA-GFP knock-in DCs migrating into 5 × 5 μm microchannels coated with 20 μg ml^−1^ fibronectin were imaged using a spinning disk microscope consisting of Yokagawa CSU-X1 spinning head mounted on an Eclipse Ti inverted microscope (Nikon) equipped with a Coolsnap HQ2 camera (Photometrics) with a × 60 differential interference contrast 0.75 NA taking one image every 10 s. Only the cell middle plan was imaged. Macropinosome velocity was measured over 2 min post formation by detection of its initial and final positions. During the same time frame, myosin IIA enrichment was determined in a region surrounding macropinosomes. To take only into account myosin IIA enrichment above background, myosin IIA patches were considered as clusters bigger than 10 pixels above the mean GFP intensity of the cell front GFP cytoplasmic signal.

### Flow cytometry analysis

Cells were resuspended in staining buffer (see above). After Fc blocking with 24G2 monoclonal Ab, the cell suspension was stained for 20 min at 4 °C with the indicated antibodies. Cells were then washed three times and were resuspended in staining buffer. For intracellular staining, cells were first surface-stained as described above, and then permeabilized, fixed and stained with anti-Myh9 or rabbit serum using the Cytofix/Cytoperm kit from BD Bioscience as recommended by the manufacturer.

### Lentivirus production and infection

For short hairpin RNA (shRNA) experiments, purified pLKO.1 lentiviral plasmids carrying shRNA sequences for Ii (NM_010545.2-718s1c1-CCGGGCGTCCAATGTCCATGGATAACTCGAGTTATCCATGGACATTGGACGCTTTTTG for ShIi1 and NM_010545.2-763s1c1–CCGGCGTTACCAAGTACGGCAACATCTCGAGATGTTGCCGTACTTGGTAACGTTTTTG for ShIi2, Sigma-Aldrich) or control shRNA (SHC002–CCGGCGTGATCTTCACCGACAAGATCTCGAGATCTTGTCGGTGAAGATCACGTTTTT, Sigma-Aldrich) were used to generate lentiviral particles. Briefly, HEK293T packaging cells were co-transfected with the transfer plasmid pLKO/shRNA, the pPAX2 packaging plasmid and the pMD2G envelope plasmid (kind gifts from D. Trono, EPFL, Lausanne, Switzerland), using the GeneJuice Transfection Reagent as recommended by the manufacturer (Novagen). Virus supernatants were titered using the QuickTiter Lentivirus Titer Kit (Cell Biolabs Inc.) and were used to infect Day2-DCs at a multiplicity of 0.03 pg of p24 per cell by adding it directly to the culture. The medium was replaced on day 3 and infected cells were selected with 4 μg ml^−1^ puromycin from day 4 to day 6. Several washes were carried out during the selection process to eliminate dead cells. Infected DCs were used on day 10 for experiments.

### Transfection of BMDCs

Bone-marrow derived dendritic cells (BMDCs) were transfected using the Amaxa mouse Dendritic Cell Nucleofector Kit (Lonza), according to the manufacturer's protocol. BMDCs collected on day 6 of differentiation (3 × 10^6^) were transfected in 100 μl of Amaxa solution containing 2 μg of plasmid (empty vector: pCDNA3; Ii full length or IiL7AL17A mutant, kindly provided by Oddmund Bakke). Transfected cells were used for 6–16 h after transfection. Transfection rate was ∼40%.

### Immunofluorescence

For immunofluorescence on cells migrating in microchannels, microchannels were prepared differently: in order to easily detach the piece of PDMS after cell fixation, only the glass slide surface was activated with plasma before sticking it to PDMS. DCs were loaded in microchannels and fixed 12 h later with 4% paraformaldehyde for 20 min at room temperature. After three washes in PBS, PDMS was gently removed and cells were permeabilized in PBS 1 × +0.2 %+BSA+0.05 % saponin for 10 min and stained with antibodies. All washes and antibody dilutions were performed in the permeabilization buffer. Slides were washed, mounted and visualized on an inverted Spinning Disk Confocal Roper/Nikon microscope with a × 63 1.4 NA or a × 100 1.4 NA oil immersion objective.

### Duo-Link *in situ* detection of protein complexes

The Duo-Link *in situ* experiment was performed according to the manufacturer's recommendations (Olink Bioscience). Briefly, immature DCs were loaded in microchannels, fixed and permeabilized as described in the *Immunofluorescence* section. DCs were stained with primary antibodies in permeabilization buffer overnight at 4 °C, washed once with the permeabilization buffer, stained with AlexaFluor488-Phalloidin for 30 min and washed again before the incubation with the secondary antibody. Anti-GFP and anti-γTubulin antibodies were detected with the commercially available anti-Rabbit Plus PLA Probe. Anti-Ii antibodies were detected with anti-Rat antibodies coupled to Minus PLA Probe using the commercially available PLA Probe Maker Kit. Slides were washed, mounted with Fluoromount and visualized on an inverted Spinning Disk Confocal Roper/Nikon microscope with a × 100 1.4 NA oil immersion objective. The Duo-Link signal was quantified as the mean intensity of the overall cell on the sum projection of *z* planes with a background subtraction.

### Structured illumination microscopy

Structured illumination image acquisition was carried out using an OMX version 3 (Applied Precision-GE Healthcare, Issaquah, WA) coupled to three EMMCD Evolve cameras (Photometrics, Tucson, AZ). Multichannel image alignment was performed using ImageJ (Rasband, W.S., ImageJ, US National Institutes of Health, Bethesda, MD, USA, http://imagej.nih.gov/ij/, 1997-2012) and UnwarpJ plugin^46^. Co-localized pixels were detected using the RG2B co-localization plug in of Image J.

### Immunoblotting

DCs were lysed for 10 min in 100 mM Tris, 150 mM NaCl, 0.5% NP-40, a protease inhibitor cocktail (Roche) and a phosphatase inhibitor cocktail (Sigma). Soluble extracts (50 μg) were loaded on a 4–20% TGX gradient gel (Bio-Rad) and were transferred on a Trans-Blot Turbo PVDF/Nitrocellulose membrane (Bio-Rad). Membranes were blocked with TBS−0.05% Tween-20+5% BSA, incubated with primary antibodies (1/1,000 anti-phosho-MLC and 1/5,000 anti-α-tubulin), washed and incubated with secondary antibodies (1/5,000 horseradish peroxidase (HRP)-conjugated antibodies). Signals were revealed with the SuperSignal West Dura substrate (Thermo Scientific).

### Myosin IIA-GFP pull-down

For each condition, 5 × 10^6^ BMDCs transfected at day 6 were lysed 8 h post transfection at 4 °C in 25 mM Tris pH 7.5, 50 mM NaCl, 0.1% NP40 and a protease inhibitor cocktail (Roche) during 1 h. Lysates were incubated with 20 μl of GFP-trap beads (Chromotek) for 5 h at 4 °C under rotation, washed four times in 1 ml lysis buffer, dried and resuspended in Laemmli Buffer, loaded on a 4–20% TGX gradient gel (Bio-Rad) and transferred on a Trans-Blot Turbo PVDF/Nitrocellulose membrane (Bio-Rad). Membranes were blocked with PBS+0.05 %Tween20+5% BSA, incubated with primary antibodies (1/1,000 anti-myosin IIA HC and 1/10 homemade anti-human Ii C-term rabbit serum), washed and incubated with secondary antibodies (1/5,000 HRP-conjugated antibodies). Signals were revealed with the SuperSignal West Dura substrate (Thermo Scientific).

### CD71 pulse-chase experiments and analysis of endosomal compartments

In all, 3 × 10^5^ DCs were loaded in microchannels 10 h before the pulse-chase. First, transferrin receptors (CD71) expressed at the surface were stained at 4 °C with antibodies diluted in medium for 20 min. After three washes with medium at 4 °C, DCs were incubated at 37 °C for 30 min, and then fixed inside micro-channels. Immunofluorescence was then performed as described above (see Immunofluorescence section) using phalloïdin-AF546, Hoechst and endosomal markers: anti-ClathrinHC, anti-EEA1, anti-Rab11, anti-Rab7, anti-Lamp1 and phalloïdin. Images were acquired on an epifluorescence Nikon microscope (Eclipse 90i Upright) with a × 100 objective. Deconvolution was performed on stacks of images taken with a 100-nm *z* section, using the 3D deconvolution module of METAMORPH and the fast iterative constrained point spread function-based algorithm^47^. Quantifications were performed using Image J. Phalloïdin staining was used as a mask for the cell. For endosomal and CD71 staining, foci were detected by applying a threshold and object segmentation by maxima.

### Analysis of lysosomal pH

AlexaFluor-488 OVA was coupled to pHRhodo Red SE, which fluoresces below pH=6.5 and is quantitatively sensitive to acidic pH. Coupling was performed following the manufacturer's manual (Life Technologies). Overall, 10^6^ DCs were incubated with 0.2 mg of pHRhodo Red dye coupled to AF488-OVA in 1 ml of the medium for 1 h at 37 °C. After three washes with medium, DCs were loaded into microchannels with 25 nM Concanamycin A or DMSO. After 6 h, cells were recorded every 3 min on an epifluorescence video-microscope Nikon TiE microscope equipped with a × 20 objective with a cooled CCD camera (HQ2, Photometrics). The relative lysosomal pH was determined as the ratio of pHRhodo Red/AF488-OVA mean intensities. For each cell, a mean ratio on five frames was calculated.

## Additional information

**How to cite this article:** Chabaud, M. *et al*. Cell migration and antigen capture are antagonistic processes coupled by myosin II in dendritic cells. *Nat. Commun.* 6:7526 doi: 10.1038/ncomms8526 (2015).

## Supplementary Material

Supplementary Information Supplementary Figures 1-7, Supplementary Methods and Supplementary References

Supplementary Movie 1 Low-resolution dynamics of myosin IIA-GFP in *WT*, *Ii*^*−/−*^ and *CatS*^*−/−*^ immature DCs migrating in micro-channels. Images were acquired on an epifluorescence microscope every 30 s (20X objective).

Supplementary Movie 2 3D projections and orthogonal views of an immature DC in a micro-channel fixed and stained for F-actin, myosin II HC and Ii. Co-localized pixels are shown in yellow. Images were acquired by Structured Illumination microscopy and reconstructed.

Supplementary Movie 3 Raw signal (middle plane) and iso-surface 3D reconstruction of an I-Ab-GFP^+^ (MHC II-GFP) immature DC migrating in a micro-channel filled with 10kDa AF647-Dextran. In the iso-surface reconstruction movie, a single vesicle was highlighted in blue for eye tracking. The fluorescent content of vesicles becomes brighter upon exocytosis. Images were acquired on a spinning disk microscope every 30 s (60X objective).

Supplementary Movie 4 High-resolution dynamics of myosin IIA-GFP at the cell front in *WT* and *Ii*^*−/−*^ immature DCs migrating in micro-channels. Images were acquired on a spinning disk microscope every 10 s (60X objective, middle plane).

Supplementary Movie 5 Accumulation of AF488-Ovalbumin (OVA) in CypHer5E-positive endolysosomal compartments in immature *WT*, *MyoIIA*^*−/−*^, *Ii*^*−/−*^ and *CatS*^*−/−*^ DCs migrating in micro-channels. Images were acquired on an epifluorescence microscope every min (20X objective).

## References

[b1] Sallusto F., Cella M., Danieli C. & Lanzavecchia A. Dendritic cells use macropinocytosis and the mannose receptor to concentrate macromolecules in the major histocompatibility complex class II compartment: downregulation by cytokines and bacterial products. J. Exp. Med. 182, 389–400 (1995). 7629501 10.1084/jem.182.2.389 PMC2192110

[b2] Garrett W. S. . Developmental control of endocytosis in dendritic cells by Cdc42. Cell 102, 325–334 (2000). 10975523 10.1016/s0092-8674(00)00038-6

[b3] West M. A., Prescott A. R., Eskelinen E. L., Ridley A. J. & Watts C. Rac is required for constitutive macropinocytosis by dendritic cells but does not control its downregulation. Curr. Biol. 10, 839–848 (2000). 10899002 10.1016/s0960-9822(00)00595-9

[b4] Norbury C. C. Drinking a lot is good for dendritic cells. Immunology 117, 443–451 (2006). 16556257 10.1111/j.1365-2567.2006.02335.x PMC1782244

[b5] Farache J. . Luminal bacteria recruit CD103(+) dendritic cells into the intestinal epithelium to sample bacterial antigens for presentation. Immunity 38, 581–595 (2013). 23395676 10.1016/j.immuni.2013.01.009 PMC4115273

[b6] Ng L. G. . Migratory dermal dendritic cells act as rapid sensors of protozoan parasites. PLoS Pathog. 4, e1000222 (2008). 19043558 10.1371/journal.ppat.1000222 PMC2583051

[b7] Lelouard H., Fallet M., de Bovis B., Meresse S. & Gorvel J. P. Peyer's patch dendritic cells sample antigens by extending dendrites through M cell-specific transcellular pores. Gastroenterology 142, 592–601 e593 (2012). 22155637 10.1053/j.gastro.2011.11.039

[b8] Rescigno M. . Dendritic cells express tight junction proteins and penetrate gut epithelial monolayers to sample bacteria. Nat. Immunol. 2, 361–367 (2001). 11276208 10.1038/86373

[b9] Tal O. . DC mobilization from the skin requires docking to immobilized CCL21 on lymphatic endothelium and intralymphatic crawling. J. Exp. Med. 208, 2141–2153 (2011). 21930767 10.1084/jem.20102392 PMC3182054

[b10] Thornton E. E. . Spatiotemporally separated antigen uptake by alveolar dendritic cells and airway presentation to T cells in the lung. J. Exp. Med. 209, 1183–1199 (2012). 22585735 10.1084/jem.20112667 PMC3371730

[b11] Lammermann T. . Rapid leukocyte migration by integrin-independent flowing and squeezing. Nature 453, 51–55 (2008). 18451854 10.1038/nature06887

[b12] Faure-Andre G. . Regulation of dendritic cell migration by CD74, the MHC class II-associated invariant chain. Science 322, 1705–1710 (2008). 19074353 10.1126/science.1159894

[b13] Solanes P. . Space exploration by dendritic cells requires maintenance of myosin II activity by IP3 receptor 1. EMBO J. 34, 798–810 (2015). 25637353 10.15252/embj.201489056 PMC4369315

[b14] Vascotto F. . The actin-based motor protein myosin II regulates MHC class II trafficking and BCR-driven antigen presentation. J. Cell Biol. 176, 1007–1019 (2007). 17389233 10.1083/jcb.200611147 PMC2064085

[b15] Hung W. C. . Distinct signaling mechanisms regulate migration in unconfined versus confined spaces. J. Cell Biol. 202, 807–824 (2013). 23979717 10.1083/jcb.201302132 PMC3760608

[b16] Zhang Y. . Mouse models of MYH9-related disease: mutations in nonmuscle myosin II-A. Blood 119, 238–250 (2012). 21908426 10.1182/blood-2011-06-358853 PMC3251230

[b17] Irimia D., Charras G., Agrawal N., Mitchison T. & Toner M. Polar stimulation and constrained cell migration in microfluidic channels. Lab. Chip 7, 1783–1790 (2007). 18030401 10.1039/b710524j PMC3001245

[b18] Gupton S. L. & Waterman-Storer C. M. Spatiotemporal feedback between actomyosin and focal-adhesion systems optimizes rapid cell migration. Cell 125, 1361–1374 (2006). 16814721 10.1016/j.cell.2006.05.029

[b19] Soderberg O. . Direct observation of individual endogenous protein complexes *in situ* by proximity ligation. Nat. Methods 3, 995–1000 (2006). 17072308 10.1038/nmeth947

[b20] Driessen C. . Cathepsin S controls the trafficking and maturation of MHC class II molecules in dendritic cells. J. Cell Biol. 147, 775–790 (1999). 10562280 10.1083/jcb.147.4.775 PMC2156161

[b21] Bremnes B., Madsen T., Gedde-Dahl M. & Bakke O. An LI and ML motif in the cytoplasmic tail of the MHC-associated invariant chain mediate rapid internalization. J. Cell Sci. 107, 2021–2032 (1994). 7983165 10.1242/jcs.107.7.2021

[b22] Koivusalo M. . Amiloride inhibits macropinocytosis by lowering submembranous pH and preventing Rac1 and Cdc42 signaling. J. Cell Biol. 188, 547–563 (2010). 20156964 10.1083/jcb.200908086 PMC2828922

[b23] Falcone S. . Macropinocytosis: regulated coordination of endocytic and exocytic membrane traffic events. J. Cell Sci. 119, 4758–4769 (2006). 17077125 10.1242/jcs.03238

[b24] Milasta S. . The sustainability of interactions between the orexin-1 receptor and beta-arrestin-2 is defined by a single C-terminal cluster of hydroxy amino acids and modulates the kinetics of ERK MAPK regulation. Biochem. J. 387, 573–584 (2005). 15683363 10.1042/BJ20041745 PMC1134986

[b25] Yuseff M. I. . Polarized secretion of lysosomes at the B cell synapse couples antigen extraction to processing and presentation. Immunity 35, 361–374 (2011). 21820334 10.1016/j.immuni.2011.07.008

[b26] Benichou O., Loverdo C., Moreau M. & Voituriez R. Intermittent search strategies. Rev. Mod. Phys. 83, 81 (2011). 10.1103/PhysRevE.80.031146 19905101

[b27] Benichou O., Coppey M., Moreau M., Suet P. H. & Voituriez R. Optimal search strategies for hidden targets. Phys. Rev. Lett. 94, 198101 (2005). 16090215 10.1103/PhysRevLett.94.198101

[b28] Benichou O., Loverdo C., Moreau M. & Voituriez R. Optimizing intermittent reaction paths. Phys. Chem. Chem. Phys. 10, 7059–7072 (2008). 19039339 10.1039/b811447c

[b29] Loverdo C., Benichou O., Moreau M. & Voituriez R. Enhanced reaction kinetics in biological cells. Nat. Phys. 4, 137 (2008).

[b30] Hawkins R. J. . Pushing off the walls: a mechanism of cell motility in confinement. Phys. Rev. Lett. 102, 058103 (2009). 19257561 10.1103/PhysRevLett.102.058103

[b31] Verkhovsky A. B., Svitkina T. M. & Borisy G. G. Myosin II filament assemblies in the active lamella of fibroblasts: their morphogenesis and role in the formation of actin filament bundles. J. Cell Biol. 131, 989–1002 (1995). 7490299 10.1083/jcb.131.4.989 PMC2200006

[b32] Veltman D. M., Lemieux M. G., Knecht D. A. & Insall R. H. PIP3-dependent macropinocytosis is incompatible with chemotaxis. J. Cell Biol. 204, 497–505 (2014). 24535823 10.1083/jcb.201309081 PMC3926956

[b33] Araki N., Hatae T., Furukawa A. & Swanson J. A. Phosphoinositide-3-kinase-independent contractile activities associated with Fcgamma-receptor-mediated phagocytosis and macropinocytosis in macrophages. J. Cell Sci. 116, 247–257 (2003). 12482911 10.1242/jcs.00235

[b34] Olazabal I. M. . Rho-kinase and myosin-II control phagocytic cup formation during CR, but not FcgammaR, phagocytosis. Curr. Biol. 12, 1413–1418 (2002). 12194823 10.1016/s0960-9822(02)01069-2

[b35] West M. A. . Enhanced dendritic cell antigen capture via toll-like receptor-induced actin remodeling. Science 305, 1153–1157 (2004). 15326355 10.1126/science.1099153

[b36] Andzelm M. M., Chen X., Krzewski K., Orange J. S. & Strominger J. L. Myosin IIA is required for cytolytic granule exocytosis in human NK cells. J. Exp. Med. 204, 2285–2291 (2007). 17875677 10.1084/jem.20071143 PMC2118468

[b37] Ilani T., Vasiliver-Shamis G., Vardhana S., Bretscher A. & Dustin M. L. T cell antigen receptor signaling and immunological synapse stability require myosin IIA. Nat. Immunol. 10, 531–539 (2009). 19349987 10.1038/ni.1723 PMC2719775

[b38] Jacobelli J., Chmura S. A., Buxton D. B., Davis M. M. & Krummel M. F. A single class II myosin modulates T cell motility and stopping, but not synapse formation. Nat. Immunol. 5, 531–538 (2004). 15064761 10.1038/ni1065

[b39] Vicente-Manzanares M., Ma X., Adelstein R. S. & Horwitz A. R. Non-muscle myosin II takes centre stage in cell adhesion and migration. Nat. Rev. Mol. Cell Biol. 10, 778–790 (2009). 19851336 10.1038/nrm2786 PMC2834236

[b40] Jacobelli J. . Confinement-optimized three-dimensional T cell amoeboid motility is modulated via myosin IIA-regulated adhesions. Nat. Immunol. 11, 953–961 (2011). 20835229 10.1038/ni.1936 PMC2943564

[b41] Caton M. L., Smith-Raska M. R. & Reizis B. Notch-RBP-J signaling controls the homeostasis of CD8- dendritic cells in the spleen. J. Exp. Med. 204, 1653–1664 (2007). 17591855 10.1084/jem.20062648 PMC2118632

[b42] Srinivas S. . Cre reporter strains produced by targeted insertion of EYFP and ECFP into the ROSA26 locus. BMC Dev. Biol. 1, 4 (2001). 11299042 10.1186/1471-213X-1-4 PMC31338

[b43] Heuze M. L. . ASB2 targets filamins A and B to proteasomal degradation. Blood 112, 5130–5140 (2008). 18799729 10.1182/blood-2007-12-128744 PMC2597609

[b44] Weber M. . Interstitial dendritic cell guidance by haptotactic chemokine gradients. Science 339, 328–332 (2013). 23329049 10.1126/science.1228456

[b45] Heuze M. L., Collin O., Terriac E., Lennon-Dumenil A. M. & Piel M. Cell migration in confinement: a micro-channel-based assay. Methods Mol. Biol. 769, 415–434 (2011). 21748692 10.1007/978-1-61779-207-6_28

[b46] Sorzano C. O., Thevenaz P. & Unser M. Elastic registration of biological images using vector-spline regularization. IEEE Trans. Biomed. Eng. 52, 652–663 (2005). 15825867 10.1109/TBME.2005.844030

[b47] Meinel E. S. Origins of linear and nonlinear recursive restoration algorithms. J. Opt. Soc. Am. 3, 387–399 (1986).

