# Supplementary material for: Cell migration and antigen capture are antagonistic processes coupled by myosin II in dendritic cells
Source: Nat Commun. 2015 Jun 25;6:7526. doi: 10.1038/ncomms8526 (PMC4491822; doi:10.1038/ncomms8526)
Supplement: Supplementary Information — Supplementary Figures 1-7, Supplementary Methods and Supplementary References [file ncomms8526-s1.pdf]

SUPPLEMENTARY FIGURE 1

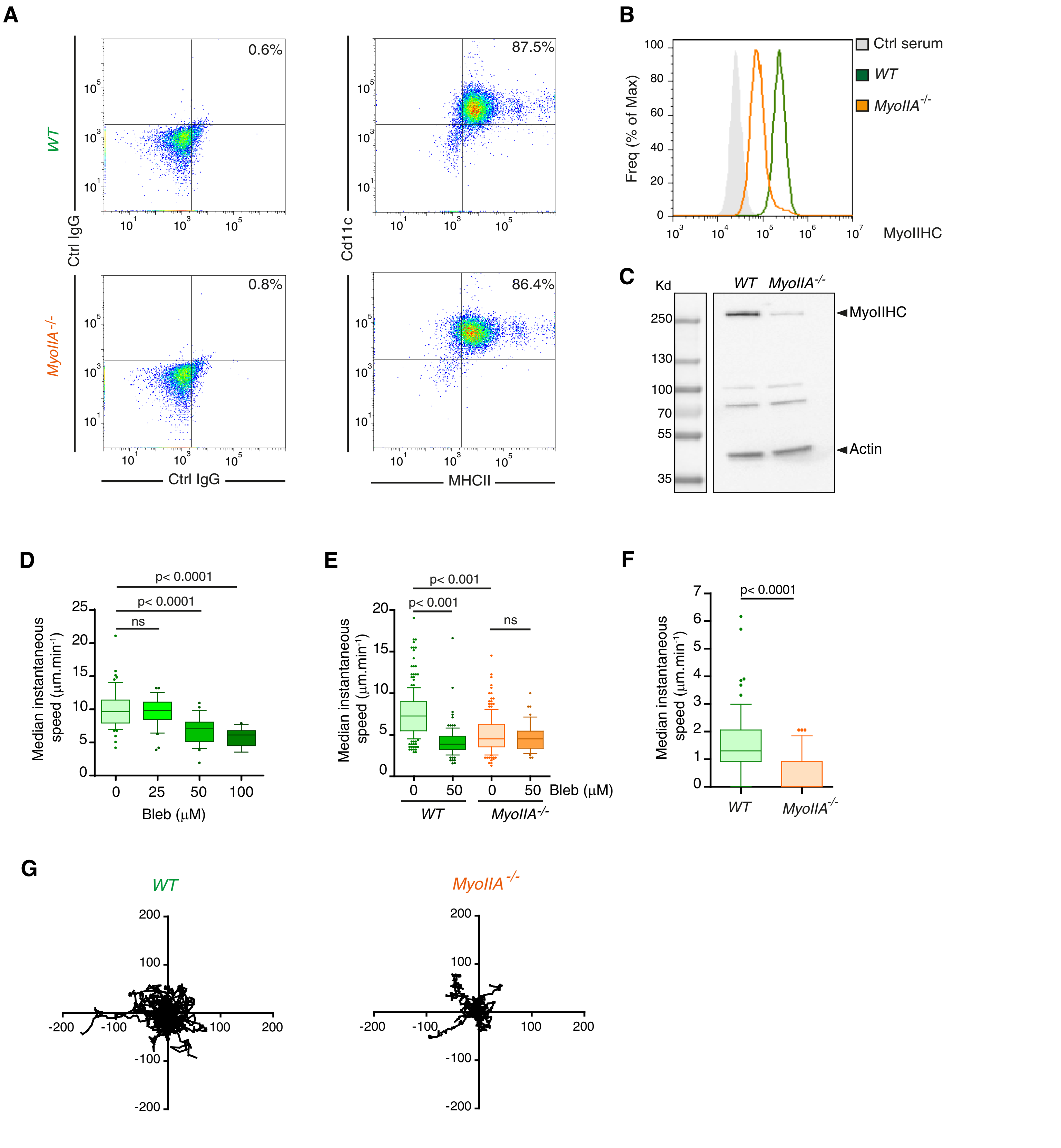

Supplementary Figure 1: Myosin IIA is required for fast migration.

A/ Dot plots from flow cytometry data showing Cd11c and MHCII surface expression in *WT* and *MyoIIA*$^{-/-}$ immature DCs. **B/** Histograms from flow cytometry data showing the intra-cellular staining for Myosin II Heavy Chain (as compared to control serum) on the Cd11c^{+}/MHCII^{+} population of *WT* and *MyoIIA*$^{-/-}$ immature DCs. **C/** Immuno-blot of total lysates obtained from *WT* and *MyoIIA*$^{-/-}$ immature DCs with an anti-Myosin II Heavy Chain antibody and an anti-actin antibody as a control. The bands at 100kD and 80kD are non specific bands detected by the anti-MyoIIAHC antibody. **D/** Median instantaneous speed of *WT* immature DCs treated with indicated concentrations of Blebbistatin . n=56, 32, 26 and 11 cells. **E/** Median instantaneous speed of *WT* and *MyoIIA-/-* immature DCs +/- 50µM Blebbistatin. n=238, 164, 104 and 48 cells from left to right. For D/ and E/, Kruskal-Wallis tests were applied for statistical analysis. **F/** Median instantaneous speed of *WT* and *Myo IIA-/-* DCs migrating in epidermal ear sheets. n=106 and 94 cells respectively, from one representative experiment out of 2. Mann-Whitney test was applied for statistical analysis.

SUPPLEMENTARY FIGURE 2

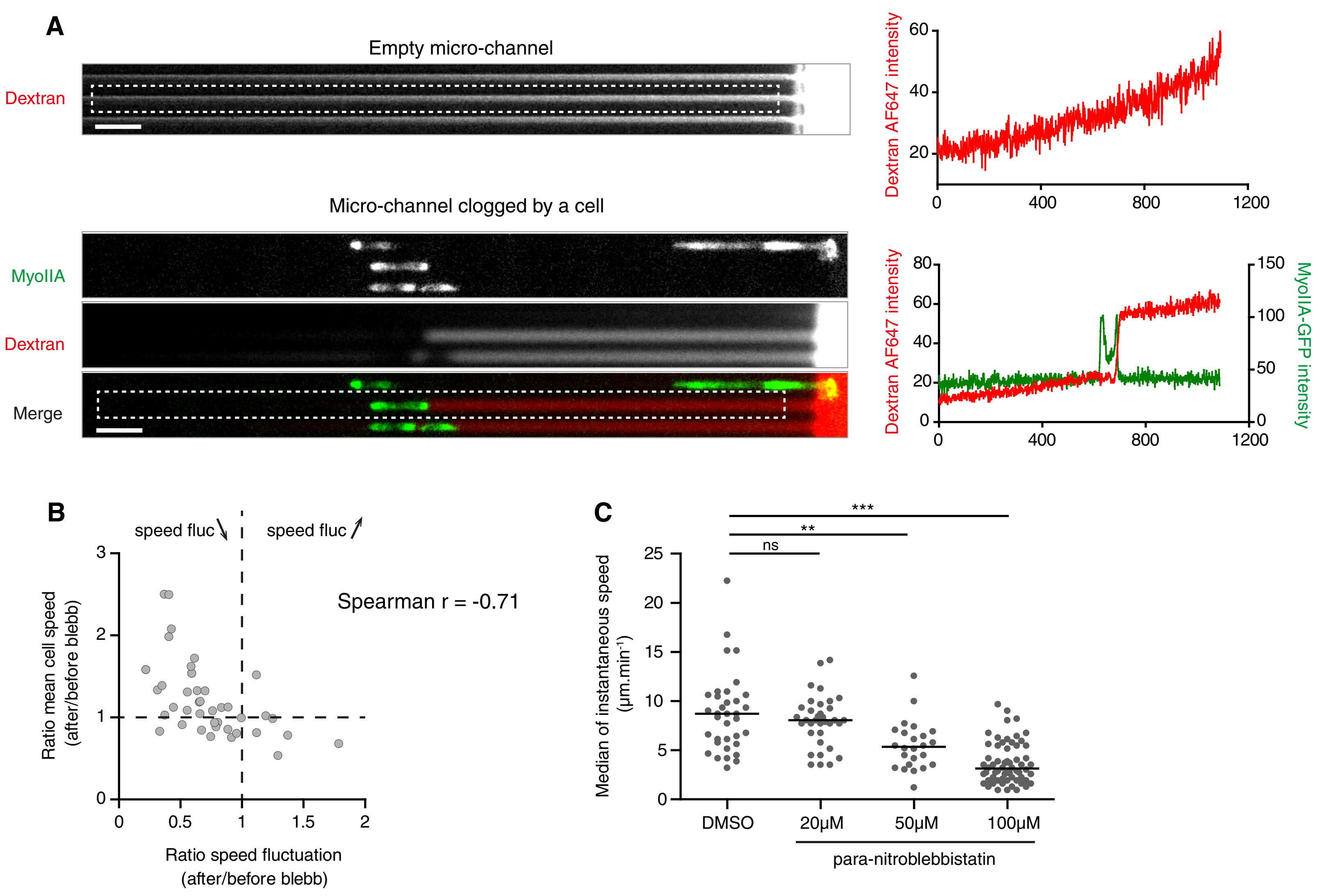

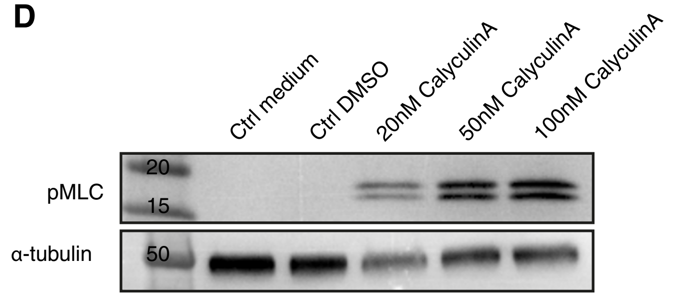

Supplementary Figure 2 : Myosin II enrichment at the front of DCs reduces their locomotion

A/ Wide field images (10X) of empty micro-channels and micro-channels clogged by *WT MyoIIA-GFP* DCs after loading AF647-dextran on one side of the micro-fluidics device. The picture and associated graphs show the gradient obtained with the micro-fluidics device when micro-channels are empty or clogged by a cell. Scale bar = 20µm. **B/** Anti-correlation between the ratio of mean instantaneous speeds and the ratio of speed fluctuations before and after treatment with 200µM Blebbistatin at the cell front (46 cells from 3 independent experiments). **C/** Median of instantaneous speed of *WT* immature DCs either treated with DMSO or with several doses of para-nitroblebbistatin (non phototoxic blebbistatin) (n=25-60 cells per condition from 1 experiment). Bars show medians. A Kruskal-Wallis test was applied for statistical analysis. **D/** Immunoblot of phospho-MLC in DCs treated or not for 40 min with several doses of calyculin A.

SUPPLEMENTARY FIGURE 3

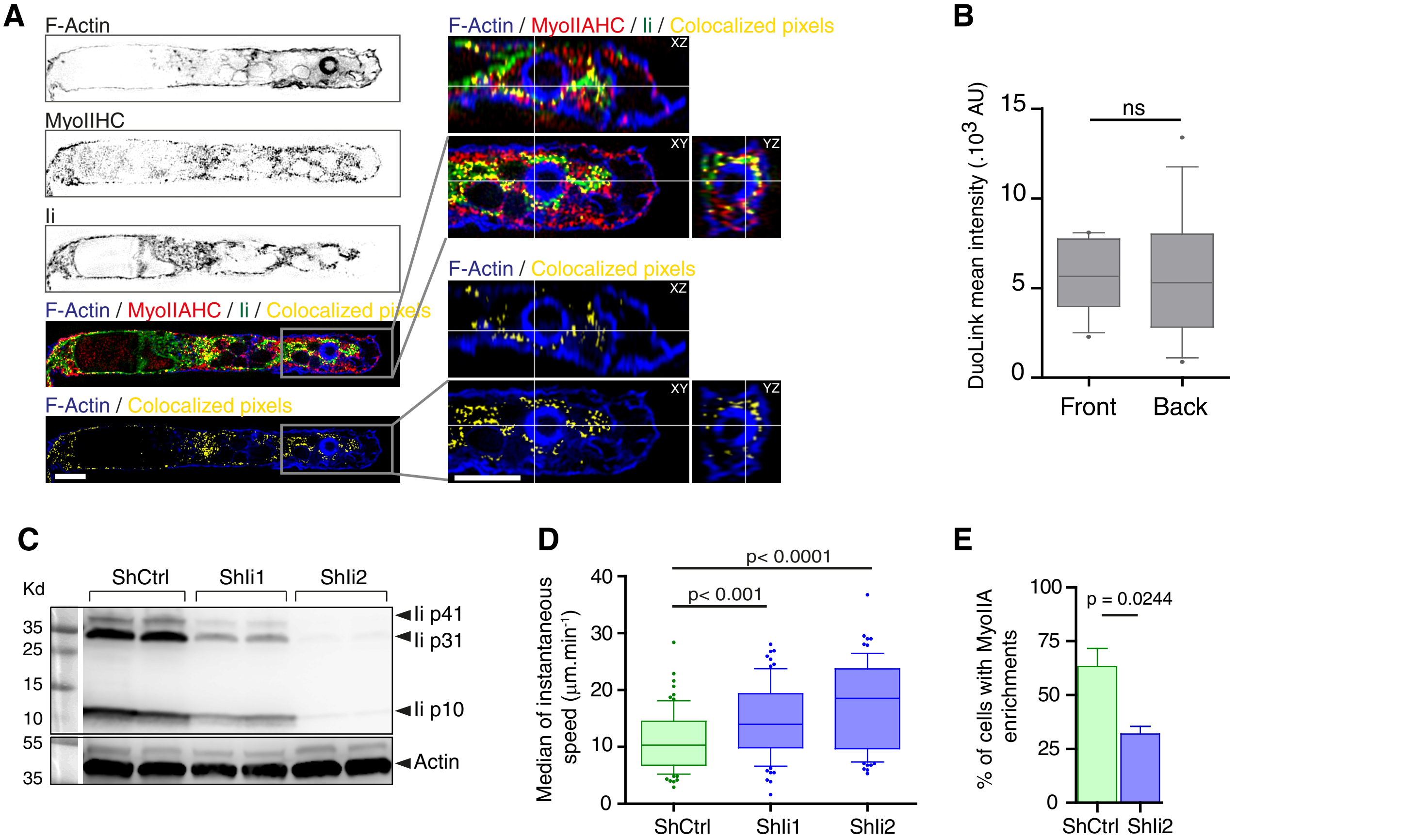

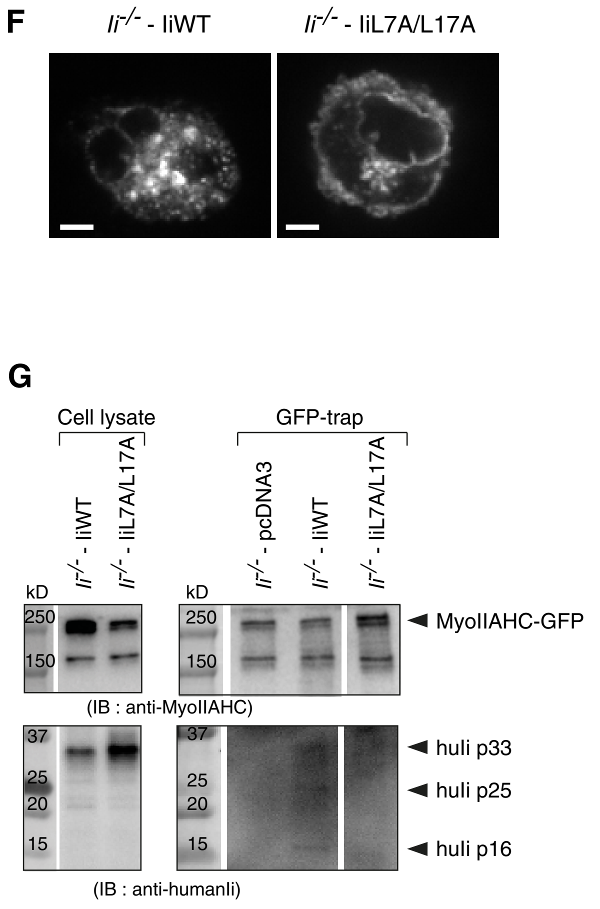

Supplementary Figure 3: Ii recruits Myosin IIA to the DC front.
A/ Single plane images acquired using SIM (Structured Illumination Microscopy) technology. Immature DCs migrating in micro-channels were fixed and immuno-stained with the indicated antibodies. Scale bar: 3µm. **B/** Quantification of the Duo-Link signal mean fluorescence intensity at the front and back of *WT Myosin IIA-GFP* cells. Data are represented as box and whiskers (10-90 percentile) plus outliers. n=14 cells from 2 independent experiments. A Mann-Whitney test was applied for statistical analysis. **C/** Immunoblot analysis of lysates obtained from DCs infected with ShCtrl, ShIi1 and ShIi2 lentivirus, using an anti-Ii antibody revealing full length p41 and p31 as well as the Ii cleavage product p10. The anti-actin antibody was used as a loading control. **D/** Median of instantaneous speeds of immature DCs infected with ShCtrl, ShIi1 and ShIi2 lentiviral particles. n=68 to 80 cells, from one representative experiment out of 2. Data are represented as box and whiskers (10-90 percentile) and outliers. Kruskal-Wallis test was applied for statistical analysis. **E/** Percentage of cells exhibiting at least one Myosin IIA enrichment at the front within 15min, represented as median +/- SEM. n=305 and 354 cells for ShCtrl and ShIi2 respectively, from 3 independent experiments. The P value was determined with a paired t-test. **F/** Single plane images of *Ii$^{-/-}$* BMDCs transfected at Day6 with full length human Ii or with human Ii mutated for the dileucines motif in N-term (L7AL17A) and immuno-stained with PIN1/BU45 antibody. Scale bars: 5µm. **G/** MyoIIA-GFP pull down in *Ii$^{-/-}$* BMDCs tranfected at D6 either with an empty vector (pCDNA3); full length human Ii (IiWT) or with human Ii mutated for the dileucines motif in N-term (IiL7A/L17A). Cell lysates were incubated with GFP-trap beads (chromotek); blots were probed with anti-MyoIIAHC antibody and anti-human Ii.

SUPPLEMENTARY FIGURE 4

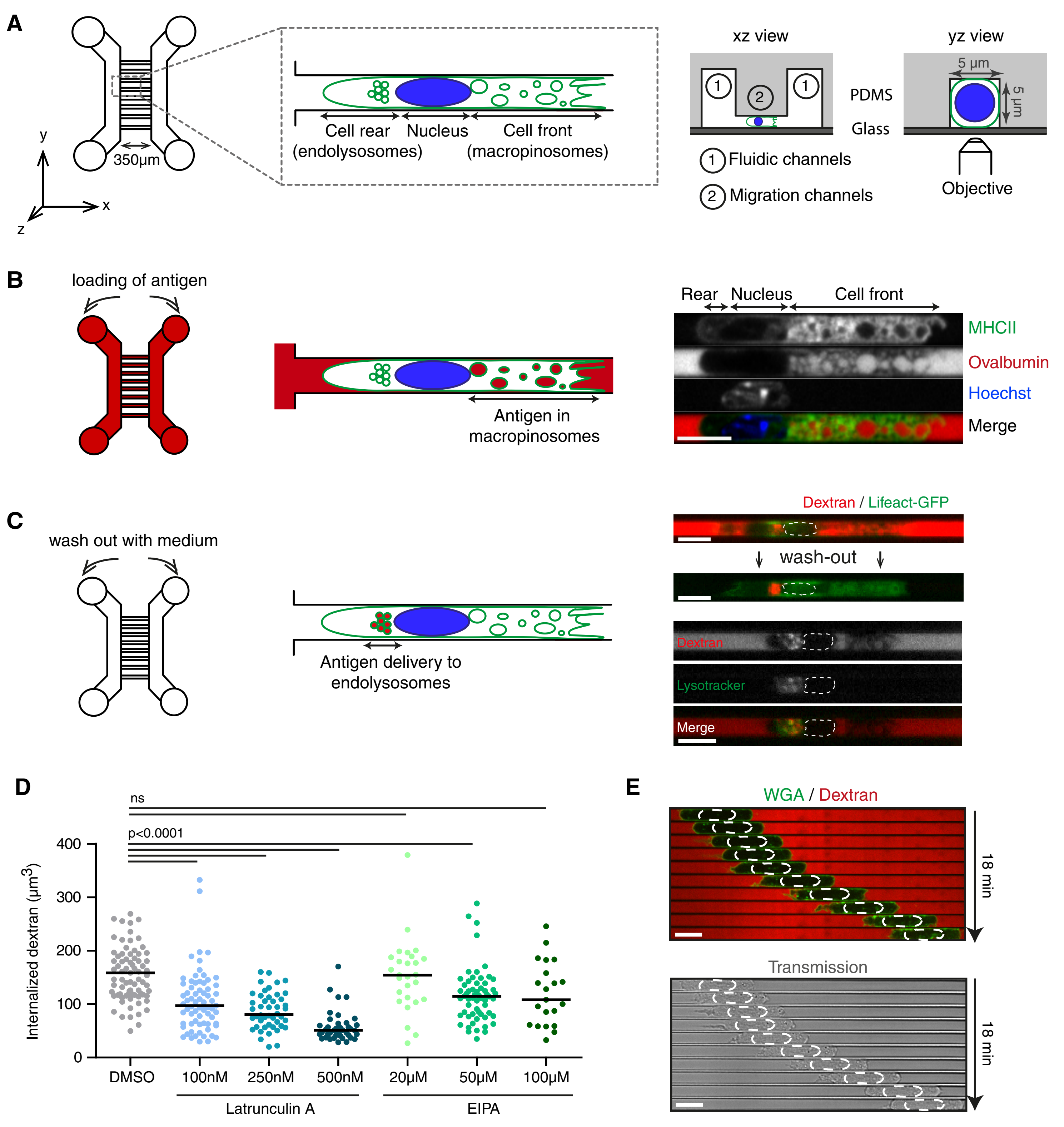

Supplementary Figure 4 : Micro-fluidics device allowing visualization of the sequential steps leading to efficient antigen internalization

A/ (Left) Scheme of the micro-fluidics device into which DCs enter and migrate spontaneously. (Right) xz and yz view of the device comprising migration channels (5x5µm) in between two fluidic channels. **B/** (Left) Addition of fluorescent antigen into fluidic channels to monitor antigen uptake. (Right) Spinning disk image (60X, middle plan) of a live *I-Ab-GFP* immature DC migrating into a micro-channel filled with 10kDa AF647-OVA. The nucleus is stained with Hoechst. **C/** (Left) Wash out fluorescent antigen with medium to monitor antigen retained inside the cell. (Right) Wide field images (20X) of a *Lifeact-GFP* immature DC before and after wash out. The compartment retaining the antigen correspond to endolysosomes as it appears lysotracker positive. **D/** Volume of 10kDa AF647-dextran internalized by immatures DCs migrating into micro-channels treated either with DMSO or several doses of Latrunculin A or EIPA. We observed high mortality at 100µM EIPA. (n=20-80 cells per condition from 2 independent experiments. A Kruskal-Wallis test was applied for statistical analyses). **E/** Sequential images (60X, middle plane) of a neutrophil stained with AF488-WGA for membrane visualization migrating along micro-channels filled with 10kDa AF647-dextran. Scale bars = 10µm.

SUPPLEMENTARY FIGURE 5

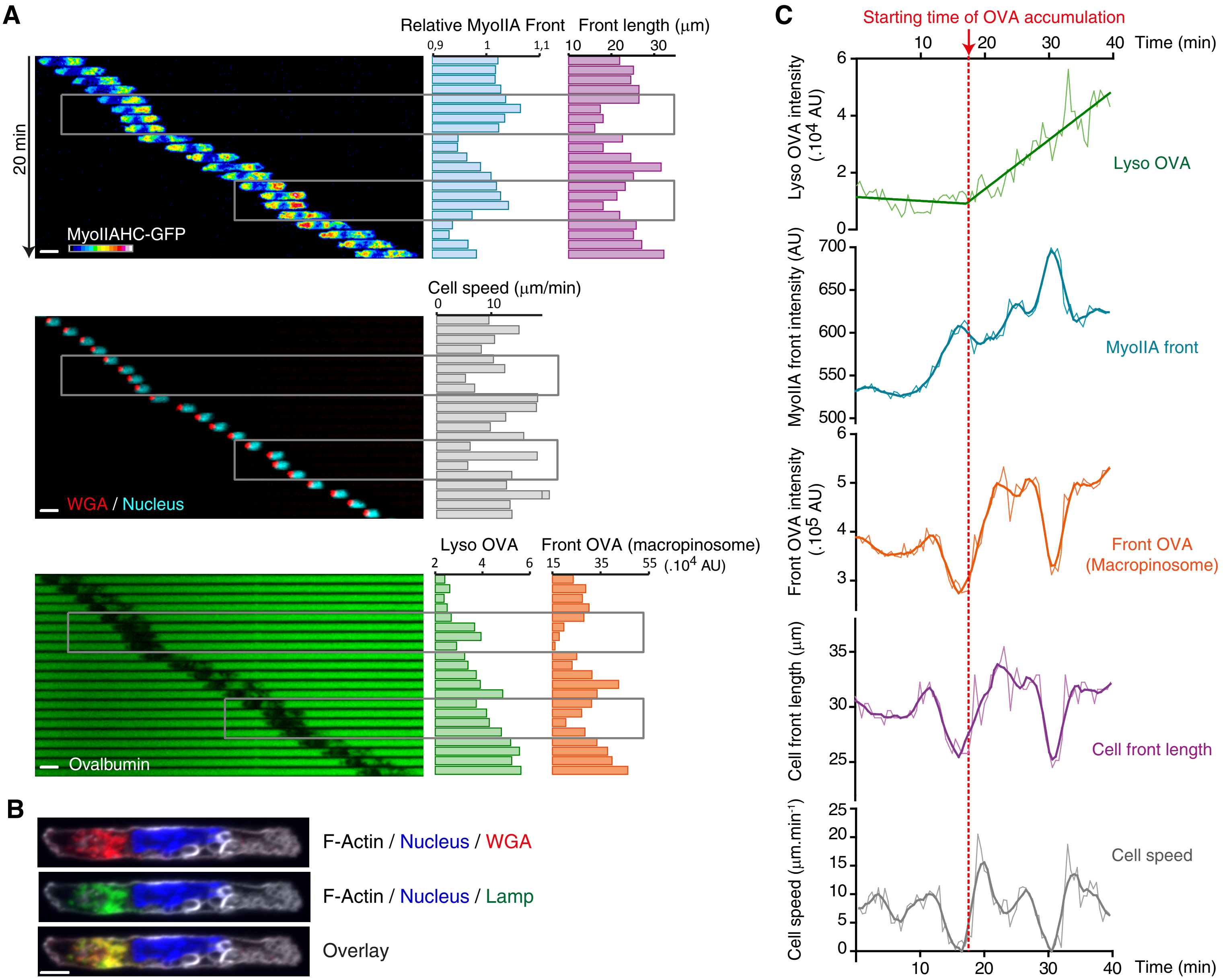

Supplementary Figure 5 : Myosin IIA enables antigen transport from macropinosomes to endolysosomes
A/ Sequential epifluorescence images (20X) of a *Myosin IIA-GFP* DC stained with Hoechst and AF555-WGA. AF647-OVA added to micro-channels just before acquisition. Four parameters are plotted over time: the average intensity of Myosin IIA-GFP at the front relative to the entire cell, the front length, the instantaneous speed of the cell nucleus, the total intensity of AF647-OVA at the front and in the WGA mask. Scale bar: 10 μm. **B/** Spinning Disk images (100X, middle plane) of fixed immature *WT* DCs stained with AF555-WGA before fixation. WGA-stained compartments overlap with Lamp-positive compartments. Scale bar: 5μm. **C/** Single cell analysis of the indicated parameters over time after addition of AF647-OVA in the channels. The mean curves measured on several cells are plotted in Fig.6B. The starting time of OVA accumulation in endolysosomes was determined using a bi-phasic non linear regression curve (dark green). Dark curves correspond to smooth fits of raw data.

SUPPLEMENTARY FIGURE 6

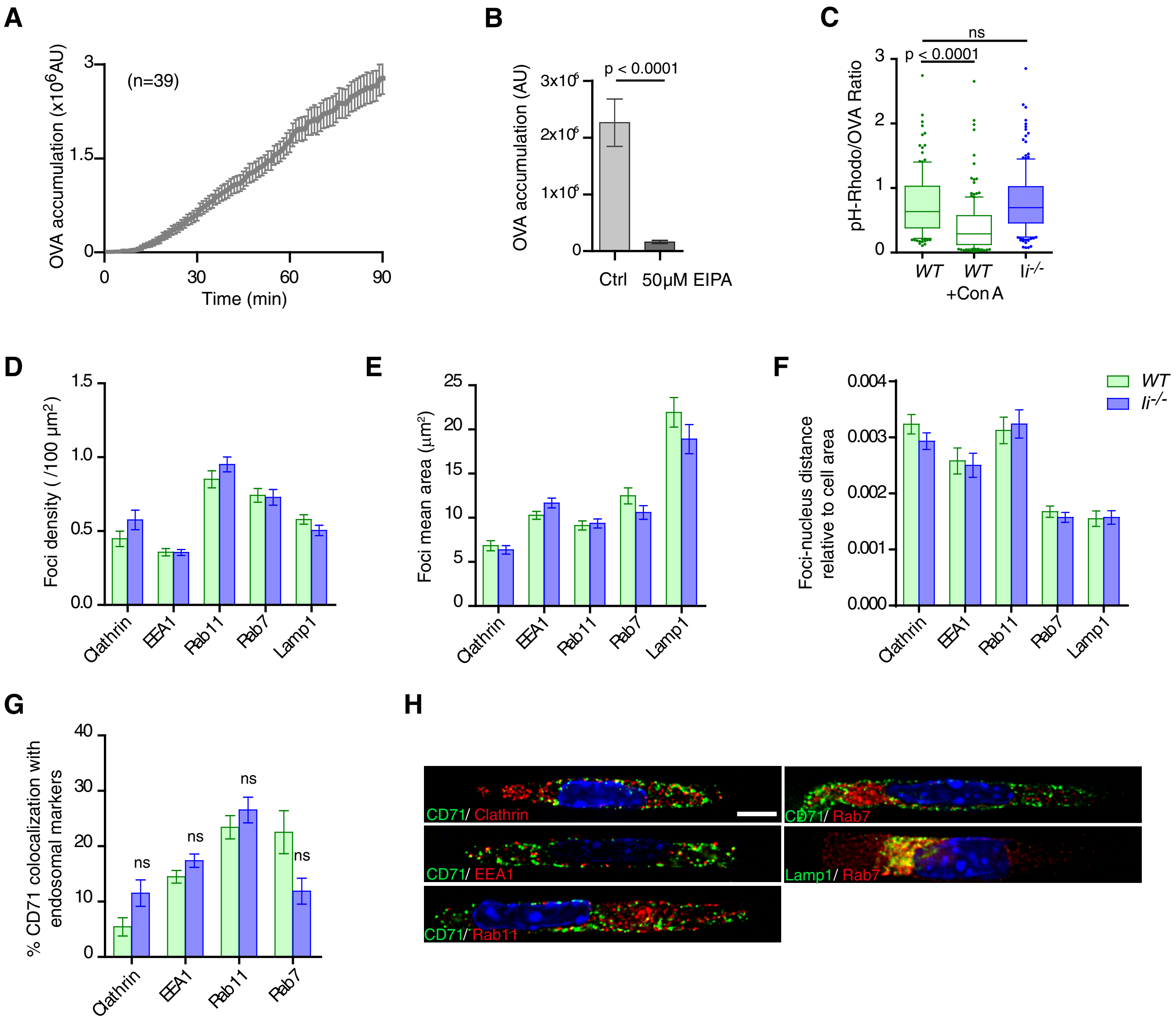

Supplementary Figure 6: Antigen transport from macropinosomes to endolysosomes

A/ AF488-OVA sum intensity into CypHer5E compartments tracked over time, representated as mean +/- SEM. Data are from 1 representative experiment out of 3. **B/** Quantification of AF488-OVA accumulation into CypHer5E positive compartments after 90 of DCs migration in micro-channels filled with coupled AF488-OVA-CypHer5E +/- 50µM EIPA. Data are represented as mean +/- SEM. n=9 and 19 cells for control and EIPA-treated cells respectively. P value was determined with a Mann-Whitney test. **C/** pH-Rhodo mean intensity relative to AF488-OVA mean intensity in *WT* +/- 50nM ConcanamycinA and *Ii-/-* DCs after 6h of migration in micro-channels. n=135, 161 and 163 cells respectively from 2 independent experiments. P value was determined with Kruskall-Wallis test. **D/ E/ F/** Analysis of endosomal compartments in *WT* and *Ii-/-* DCs fixed in micro-channels; (D) foci density, (E) foci mean area and (F) mean distance between foci and nucleus normalized by the cell area. **G/** Percentage of CD71 staining colocalizing with endosomal markers. *WT* and *Ii-/-* in DCs migrating in micro-channels pulsed with anti-CD71 and chased for 30 minutes before fixation. (n= 30 *WT* and 70 *Ii-/-* cells from 2 independent experiments. Data are represented as mean +/- SEM. No significant differences were detected using Kruskall-Wallis test. **H/** Deconvoluted epifluorescence images (100X) of single midle planes for indicated stainings performed after anti-CD71 pulse-chase. Bar: 10µm.

SUPPLEMENTARY FIGURE 7

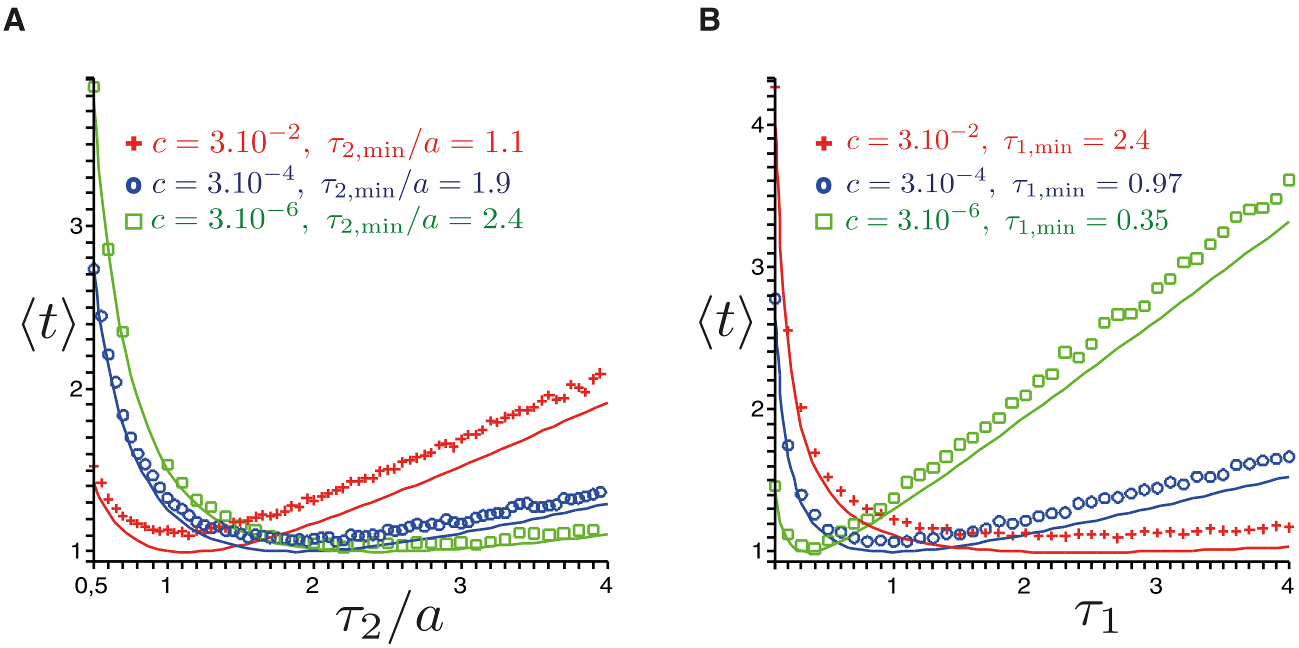

Supplementary Figure 7: Migratory behavior of DCs as an intermittent search strategy.

A/ Mean search time for an antigen as a function of the scaled time τ_2/a for different antigen concentrations, with v=1,k=1, b=56 (units are arbitrary). Analytic predictions (plain lines) are compared to numerical simulations (symbols). The optimal search strategy, defined as the minimum of the mean search time is characterized by $\tau_{1,min}$ and $\tau_{2,min}$. It is given for each concentration, and is well predicted by Eq.(3). **B/** Mean search time for an antigen as a function of the time τ_1 for different antigen concentrations, with v=1,k=1, b=56 (units are arbitrary). Analytic predictions (plain lines) are compared to numerical simulations (symbols).

Supplementary Methods: physical model for the migratory behavior of DCs as an intermittent search strategy

The data presented in this paper show that immature DCs migrate by alternating high motility phases with low motility phases during which macropinosomes are resorbed and their content transported to endolysosomes promoting antigen retrieval. We suggest here that such biphasic mode of locomotion belongs to the class of “intermittent random walks [1-4]. The intermittent random walk model relies on the assumption that efficient probing of the environment by an agent looking for a target is incompatible with a fast motion: search trajectories are in that case characterized by an alternation of slow motion with high detection abilities, and fast motion with reduced detection. The analysis of the intermittent random walk model then demonstrates on general grounds that adjusting the durations of the slow and fast phases can minimize the search time for randomly hidden targets [3]. We here briefly recall the intermittent random walk model in its minimal version, as depicted in Figure 8A. We assume that a DC evolves in a 2-dimensional environment, where antigen are present with effective concentration $c=a^2/b^2$ where a is the detection radius and b is the typical distance between antigen locations. The DC alternates slow phases (i) during which we assume that antigen capture occurs with rate k within the detection radius a, and fast phases (ii) during which we assume that antigen uptake occurs with rate $k' \ll k$. The mean duration of a phase (i) (resp. (ii)) is denoted by $\tau 1$ (resp. $\tau 2$); the velocity in phase (ii) has a random direction and modulus v and motion in phase (i) is neglected in a first approximation. Following Benichou et al., 2011[3], the efficiency of the search process is quantified by the mean search time $<t>$ for a target, which is then given analytically by:

$$\langle t\rangle = \frac{\tau_1 + \tau_2}{2k\tau_1 y^2}\left\{\frac{1}{x}(1 + k\tau_1)(y^2 - x^2)^2 \frac{I_0(x)}{I_1(x)} + \frac{1}{4}\left[8y^2 + (1 + k\tau_1)\left(4y^4 \ln(y/x) + (y^2 - x^2)(x^2 - 3y^2 + 8)\right)\right]\right\} \quad (1)$$

where I_k denote modified bessel functions and

$$x = \sqrt{\frac{2k\tau_1}{1 + k\tau_1}\frac{a}{v\tau_2}} \qquad y = \sqrt{\frac{2k\tau_1}{1 + k\tau_1}\frac{b}{v\tau_2}}. \quad (2)$$

The mean search time <*t*> can then be shown (in the limit of low antigen concentration) to be minimized for the following values of the mean durations τ1 and τ2 of the slow and fast phases:

$$\begin{aligned} \tau_{1,\min} &= \left(\frac{a}{vk}\right)^{1/2}\left(\frac{2\ln(b/a) - 1}{8}\right)^{1/4}, \\ \tau_{2,\min} &= \frac{a}{v}\left(\ln(b/a) - 1/2\right)^{1/2}, \end{aligned} \quad (3)$$

Equation (3) defines the optimal search strategy of the model. Figure S7 shows that the analytical prediction of Eq. (1) is in very good agreement with numerical simulations. In particular, the mean search time for a target antigen can be clearly minimized as a function of τ1 and τ2. While the model is presented here in its minimal version, qualitatively similar results can be obtained under much less restrictive hypothesis [3].

Supplementary References

1 *Benichou, O., Coppey, M., Moreau, M., Suet, P. H. & Voituriez, R. Optimal search strategies for hidden targets. Phys Rev Lett **94**, 198101 (2005).*

2 *Benichou, O., Loverdo, C., Moreau, M. & Voituriez, R. Optimizing intermittent reaction paths. Phys Chem Chem Phys **10**, 7059-7072 (2008).*

3 *Benichou, O., Loverdo, C., Moreau, M. & Voituriez, R. Intermittent search strategies. Rev Mod Phys **83**, 81 (2011).*

4 *Loverdo, C., Benichou, O., Moreau, M. & Voituriez, R. Enhanced reaction kinetics in biological cells. Nat Phys **4**, 137 (2008).*
